# Abnormal Hair Development and Apparent Follicular Transformation to Mammary Gland in the Absence of Hedgehog Signaling

**DOI:** 10.1016/j.devcel.2006.12.006

**Published:** 2007-01

**Authors:** Amel Gritli-Linde, Kristina Hallberg, Brian D. Harfe, Azadeh Reyahi, Marie Kannius-Janson, Jeanette Nilsson, Martyn T. Cobourne, Paul T. Sharpe, Andrew P. McMahon, Anders Linde

**Affiliations:** 1 Department of Oral Biochemistry, Sahlgrenska Academy at Göteborg University, Medicinaregatan 12F, Göteborg, Sweden; 2 Department of Molecular Genetics and Microbiology, University of Florida College of Medicine, 1600 SW Archer Road, Gainesville, FL 32610, USA; 3 Department of Cell and Molecular Biology, Göteborg University, Göteborg, Sweden; 4 Dental Institute, Department of Craniofacial Development, King's College London, Guy's Hospital, London Bridge, United Kingdom; 5 Department of Molecular and Cellular Biology, The Biolabs, Harvard University, 16 Divinity Avenue, Cambridge, MA 02138, USA

**Keywords:** DEVBIO

## Abstract

We show that removing the Shh signal tranducer Smoothened from skin epithelium secondarily results in excess Shh levels in the mesenchyme. Moreover, the phenotypes we observe reflect decreased epithelial Shh signaling, yet increased mesenchymal Shh signaling. For example, the latter contributes to exuberant hair follicle (HF) induction, while the former depletes the resulting follicular stem cell niches. This disruption of the niche apparently also allows the remaining stem cells to initiate hair formation at inappropriate times. Thus, the temporal structure of the hair cycle may depend on the physical structure of the niche. Finally, we find that the ablation of epithelial Shh signaling results in unexpected transformations: the follicular outer root sheath takes on an epidermal character, and certain HFs disappear altogether, having adopted a strikingly mammary gland-like fate. Overall, our study uncovers a multifaceted function for Shh in sculpting and maintaining the integrity and identity of the developing HF.

## Introduction

**Figure 1:**
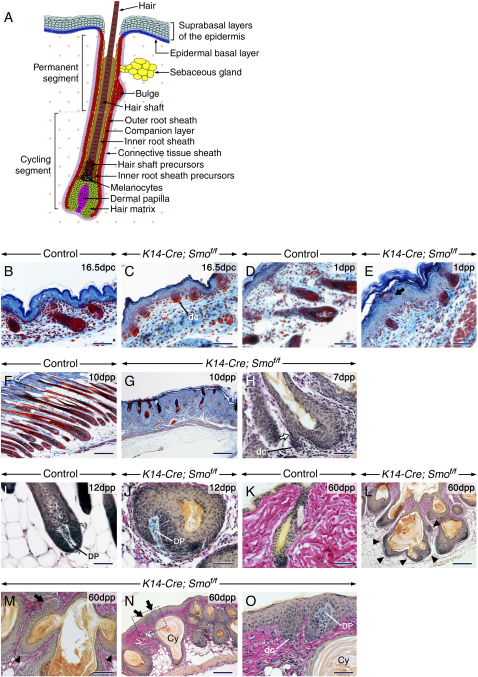
Abnormal Hair Follicle Morphogenesis and De Novo Hair Follicle Development in the Absence of Epithelial Smo Activity (A) Schematic of the histology of a mature hair follicle (HF). The hair matrix consists of proliferating cells (green) encasing the dermal papilla (magenta). Upon differentiation, matrix cells produce the concentric rings of cells that generate the hair shaft (dark brown) and inner root sheath (IRS; light brown). The upper matrix contains melanocytes (black). The outer root sheath (ORS; red) is continuous with the basal layer (blue) of the epidermis. The companion layer (orange) separates the ORS from the IRS. The ORS contains follicular stem cells nested in the bulge niche underneath the sebaceous gland (yellow). The HF is surrounded by connective tissue sheath cells (CTS; violet). Dermal cells are shown as dots. In contrast to the HF permanent segment, the cycling lower segment undergoes phases of growth and degeneration. (B–O) Skin sections from control (B, D, F, I, and K) and *K14-Cre; Smo^f/f^* mutant (C, E, G, H, J, and L–O) mice stained with Ladewig's (B–G) or Alcian Blue-van Gieson (H–O) stains. (E) Ectopic follicular cells among epidermal keratinocytes (arrow). (H and J) The de novo follicles that formed from pre-existing follicles (arrow) are underlain by dermal condensates (dc) or encase a dermal papilla (DP). (L–M) Numerous de novo-generated HF from a pre-existing follicle (arrowheads in [L] and [M]), from a pre-existing de novo-formed HF (arrow in [M]), and from the epidermis (arrows in [N]). (O) is a high-magnification view of the indicated area in (N). Cy, cyst. Scale bars: 50 μm (B–E, H–K, and O), 100 μm (M), and 200 μm (F, G, and L).

The mature HF is a complex organ (Figure 1A). Postnatally, it consists of an ectodermally derived hair shaft surrounded in its sub-epidermal portion by an inner root sheath (IRS). The shaft and IRS are formed through terminal differentiation of transiently proliferating cells of the hair matrix. The outer root sheath (ORS) encases the entire HF and is continuous with the basal layer of the epidermis. The companion layer separates the IRS from the ORS. The HF mesenchyme consists of a dermal papilla (DP), which is crucial for proliferation of matrix cells (Jahoda and Reynolds, 1993), and the connective tissue sheath (CTS). The epithelium of a follicle cycles through sequential phases of rapid growth (anagen), apoptotic regression (catagen), and quiescence (telogen). In response to signals from the DP, stem cells located in a nested area of the ORS, the bulge, give rise to transiently proliferating keratinocytes (Costarelis et al., 1990).

A myriad of molecules belonging to several signaling pathways interact in a tightly controlled manner to assist HF formation (Schmidt-Ullrich and Paus, 2005). Sonic hedgehog (Shh) is crucial for hair development and cycling, and the deregulated function of members of the Shh signaling cascade alters HF formation and generates epidermal neoplasia (McMahon et al., 2003).

Lack of Shh impinges upon HF growth and morphogenesis (St-Jacques et al., 1998, Chiang et al., 1999). Whether this is a primary defect intrinsic to the epithelium or results from abnormal epithelial-mesenchymal interaction could not be addressed in that model. Despite strong evidence suggesting a major role for epithelial Gli2 in controlling HF morphogenesis, some types of HF develop normally in *Gli2^−/−^* skin, indicating different requirements for Gli2 function depending on HF type (Mill et al., 2003). The conditional ablation of *Smoothened* (Smo; an obligatory component for all Hedgehog signaling) in the HF epithelium allows this issue to be investigated. In contrast to *Gli2^−/−^* skin (Mill et al., 2003), our findings indicated that loss of epithelial Smo activity leads to severely altered growth, morphogenesis, and differentiation of all HF and to hyperplasia of the interfollicular epidermis (IFE). Further, dorsal and ventral mesenchymal cells have been shown to differ not only in their embryonic origin but also in their ability to respond to environmental cues (Mauger, 1972, Lowe et al., 2000). We have examined these differences with respect to Hedgehog (Hh) signaling following epithelial Smo removal. Finally, we provide insight into the consequences of absence of Shh signaling on the follicular stem cell niche.

## Results

### Removal of Smo Activity in the Ectoderm and Its Derivatives

To determine the specific role of Shh in the epithelium of the developing HF, we abrogated Shh responsiveness in skin keratinocytes by K14-Cre-mediated inactivation of a conditional *Smo* allele (*floxed; [K14-Cre; Smo ^f/f^]*) or by Shh-dependent removal using a *Shhgfp-Cre* knockin allele. To visualize cells that underwent Cre-mediated recombination at the *R26R* reporter locus as well as their progeny, we used β-galactosidase histochemistry in skin from *K14-Cre; R26R* and *Shhgfp-Cre; R26R* mice. The *K14-Cre; R26R* skin displayed robust *lacZ* staining both during embryogenesis and postnatally in all cells of the epidermis and pilosebaceous unit (Figures S1A–S1D [see the Supplemental Data available with this article online]), aside from neural crest-derived melanocytes (Figures S1E–S1F). In contrast to *K14-Cre; R26R* skin, β-galactosidase-stained skin sections from *Shhgfp-Cre; R26R* embryos and postnatal animals (Figures S1G–S1R) revealed a subset of *lacZ-*negative follicular cells, including a subset of sebaceous glands, ORS cells, and cells of the follicular bulge (stem cell region).

*K14-Cre; Smo^−/f^* mutant pups lacked milk in their stomach and died within 1 day of birth. By contrast, *K14-Cre; Smo^f/f^* survived at least 12 days postpartum (dpp), i.e., after birth, up to a maximum of more than 3 months. *K14-Cre; Smo^f/f^* mice were devoid of pelage hair, a fully penetrant phenotype. A complete lack of enamel resulted in severe dental wear; mutants were therefore fed pulverized food (Figure S2). *Shhgfp-Cre; Smo^f/f^*, *Shhgfp-Cre; Smo^−/f^*, *K14-Cre; Shh^−/f^*, and *K14-Cre; Shh^f/f^* mutants died 24 hr after birth. *Shh^−/−^* mutants died at birth. Conditional homozygotes without Cre activity were fertile and phenotypically similar to wild-type animals, as were mice carrying only one mutant allele (regardless of whether it was null or conditional, for *Shh* or *Smo*). These animals served as controls in our study.

### Altered Follicular Growth and Morphogenesis of HF, De Novo HF Formation, and Epidermal Hyperplasia in the Absence of Epithelial Smo Activity

At 16.5 days postcoitum (dpc), *K14-Cre; Smo^f/f^* follicles were growth-delayed. However, in contrast to *Shh^−/−^* (St-Jacques et al., 1998, Chiang et al., 1999) and to *K14-Cre; Shh^−/f^* mutants (Table S1), the dermal condensates (dc) underlying the *K14-Cre; Smo^f/f^* hair germs were well developed (Figure 1C). By 1 dpp, mutants showed abnormal epithelial invaginations associated with dc or DP instead of typical HF (Figure 1E). In pigmented animals, the early mutant HF displayed melanin granules, indicating normal melanogenesis.

The *K14-Cre; Smo^f/f^* follicles increased in size progressively and were devoid of the epithelial lamination typical of mature HF (Figure 1G). Starting at 7 dpp, epithelial buds resembling early HF as they were underlain by dc or encased a DP developed from the ORS of pre-existing follicles (Figures 1H and 1J). The number of follicles exhibiting these features as well as the size and number of follicles emanating from pre-existing ones increased progressively with age. In control mice aged 2–3 months, HF were at the resting telogen phase (Figure 1K). At this stage, the mutants displayed huge follicles, many of which exhibited as many as 5–7 new follicles that emanated from their ORS. The new follicles were associated with dc or DP and formed rudimentary hair shafts, indicating de novo folliculogenesis, i.e., late postnatal development of HF that are normally established only during embryogenesis (Figure 1L). New follicles also emerged from pre-existing de novo-formed ones. (Figure 1M). Furthermore, epithelial invaginations associated with dc/DP were detected in the IFE (Figure 1N), indicating de novo follicular formation from the IFE, with the first ones initiating at 12 dpp (data not shown). The mutant follicles converted progressively into cysts filled with cornified debris (remnants of degenerated keratinocytes).

Throughout all stages in mutants, the IFE was hyperplastic and the dermis showed hypercellularity (Figures 1B–1O). From 9 dpp, ectopic melanin-containing cells were detected in the dermis of mutants. Only a few follicles exhibited sebaceous glands.

Some of the above phenotypes, such as the failure of hair follicle morphogenesis, are consistent with known requirements for Shh signaling. However, the presence of ectopic dc/DP and associated HF, and the hyperplasia of the IFE, are phenotypes that one might have expected from increased Shh signaling rather than a loss of Shh responsiveness. We therefore entertained the hypothesis that the loss of epithelial Shh signaling, and thus epithelial Ptc expression, would lead to decreased Shh turnover and thus to increased steady-state levels of Shh available to signal to the mesenchyme.

### Altered Shh Signaling in the Absence of Smo Activity

**Figure 2:**
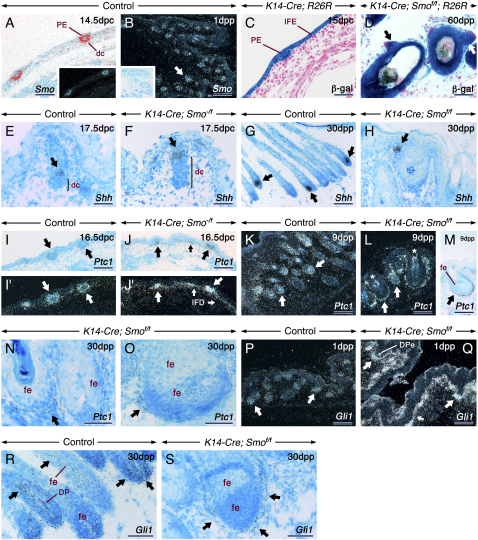
Altered Hh Signaling in the Absence of Epithelial Smo Activity: Abrogated Signaling in the HF Epithelium and Upregulated Signaling in the Skin Mesenchyme In situ hybridization of skin sections from control (A, B, E, G, I, I′, K, P, and R), *K14-Cre; Smo^−/f^* (F, J, and J′), and *K14-Cre; Smo^f/f^* (H, L–O, Q, and S) mice. β-galactosidase (β-gal) histochemistry (blue) of skin sections from a *K14-Cre; R26R* embryo (C) and a *K14-Cre; Smo^f/f^; R26R* mutant (D) showing cells that underwent Cre-mediated recombination at the *R26R* reporter locus and their progeny. (A and B) At 14.5 dpc (A), *Smo* is expressed in the hair placode (PE) and dermal condensates (dc). At 1 dpp (B) *Smo* is expressed in both the HF epithelium and mesenchyme (arrow). (C) At 14.5–15 dpc, the PE and keratinocytes of the interfollicular epidermis (IFE) are β-gal positive. (D) The mutant follicles are β-gal positive. The arrows shows de novo HF that formed from pre-existing ones (D). (E–H) *Shh* expression (arrows) in control and mutant HF. The brackets in (E) and (F) indicate the dc, which are expanded in the mutant. (H) *Shh* expression in a de novo-formed follicle from the IFE. (I–S) In control skin, *Ptc1* and *Gli1* are expressed in the follicular epithelium (fe), dc, and dermal papillae (DP) (arrows). By contrast, in the mutants, *Ptc1* and *Gli1* transcripts are virtually absent in the follicular epithelium, whereas the dc (thick arrows) and the mesenchyme underlying the IFE (thin arrows) show upregulated *Ptc1* and *Gli1* expression. (Q) *Gli1*-positive DP cells engulfed by a follicular ingrowth (DPe). Artifacts due to keratin deposits and celluar debris in the follicular epithelium are shown (asterisks in [L]). Scale bars: 50 μm (A, C, D, E, F, H, N, O, R, and S) and 100 μm (B, G, I–M, P, and Q).

*Smo* is expressed in both the epithelium and mesenchyme of HF (Figures 2A and 2B). At 14.5–15 dpc, *Smo* expression coincided with K14-Cre-mediated recombination (Figure 2C). Robust *lacZ* staining was maintained in keratinocytes of both control and mutant skin throughout all stages (Figure 2D and Figures S1A–S1F). In control HF, *Shh* was expressed in a subset of cells in the epithelium and matrix (Figures 2E and 2G). In *K14-Cre; Smo^f/f^* skin, both follicles that arised during embryogenesis and the de novo-generated ones expressed *Shh* (Figures 2F and 2H and Figure S3N). The expression of the Hh targets *Ptc1* and *Gli1* provides a direct indication of Shh responsiveness (Ingham and McMahon, 2001). To verify the effectiveness of K14-Cre-mediated removal of *Smo*, we examined the expression of *Ptc1* and *Gli1* in the mutant skin. Control HF expressed *Ptc1* and *Gli1* in both the epithelium and mesenchyme during HF morphogenesis and anagen (Figures 2I′, 2K, 2P, and 2R). By contrast, in mutants, *Ptc1* and *Gli1* expression was reduced to background levels in the follicular epithelium and was increased in the dc/DP as well as in the dermis underlying the IFE (Figures 2J′, 2L–2O, 2Q, and 2S). These findings support a model in which loss of epithelial Smo leads indirectly to increased Shh availability to signal to the mesenchyme.

### Abnormal Shaft/IRS Formation, Extrusion of Follicular Keratinocytes, and Expansion of dc/DP in the Absence of Epithelial Smo Function

To determine more accurately the extent of follicular defects and gain insight into their pathogenesis in the absence of epithelial Smo activity, we used immunohistochemistry and in situ hybridization with a set of markers. (The different markers used and their detailed expression patterns in control and mutant skins are listed in Table S2.)

In mutant skin, E-cadherin, K14, and syndecan-1 staining revealed an increased thickness of the IFE and ORS (Figures S3A–S3H). Keratin 6 (K6) staining revealed an expanded companion layer (Figures S3I–S3K). The de novo-formed follicles from pre-existing ones and from the IFE expressed a number of HF markers including *Shh*/Shh, *Bmp4*, *Msx1/2*, *Wnt5a*, and *Wnt10b* (Figures S3L–S3Q and data not shown).

**Figure 3:**
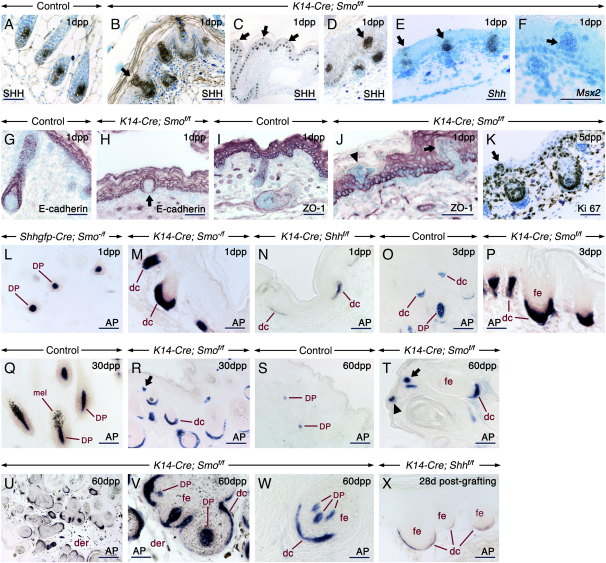
Extrusion of HF Precursors and Expansion of Dermal Condensates and Dermal Papillae in the Absence of Epithelial Smo Activity Skin sections from control (A, G, I, O, Q, and S), *K14-Cre; Smo^f/f^* (B–F, H, J, K, P, R, and T–W), *Shhgfp-Cre, Smo^−/f^* (L), *K14-Cre; Smo^−/f^* (M), and *K14-Cre; Shh^f/f^* (N). Section of a skin graft from a *K14-Cre; Shh^f/f^* fetus at 28 days postgrafting (X). Sections were processed for immunohistochemistry, in situ hybridization, or alkaline phosphatase (AP) histochemistry. The *K14-Cre; Smo^f/f^* skin exhibits follicular cells embedded in the epidermis (arrows in [B]–[F], [H], [J], and [K]) or extruded at the skin surface (arrowhead in [J]). These ectopic follicular cells express Shh/*Shh* (B–E), *Msx2* (F), and Ki 67 (K) and are devoid of E-cadherin (H) and ZO-1 (J) staining. Expansion of alkaline phosphatase-positive (blue/purple) dc/DP in *K14-Cre; Smo^−/f^* (M) and *K14-Cre; Smo^f/f^* (P, R, and T–W) skins as compared to *Shhgfp-Cre; Smo^−/f^* (L) and control (O, Q, and S) skins. Hypoplastic dc/DP in skin (N) and a skin graft (X) lacking *Shh*. dc associated with de novo-generated follicles from the interfollicular epidermis (arrows in [R] and [T]). A dc within the epidermis (arrowhead in [T]). (U–W) DP cells engulfed by the follicular epithelium (fe). Dermis (der) of a pigmented mutant skin showing numerous melanin-containing cells (U and V). (V) is a high-magnification view of (U). mel, melanocytes. Scale bars: 50 μm (A, B, D–Q, S, T, and V–X), 100 μm (R) and 200 μm (C and U).

During early postnatal stages, some Shh-positive cells formed aggregates within the epidermis or were found at the skin surface, indicating their potential extrusion (Figures 3B–3D). Aggregates of extruded matrix cells were also detected by ZO-1, E-cadherin, and Ki67 staining (Figures 3G–3K). Further, these cell aggregates expressed *Shh*, *Msx1/2*, *Bmp4*, *Epiprofin*, *Wnt5a*, and *Wnt10b* (Figures 3E and 3F, Figures S4B and S4J, and data not shown), indicating their follicular nature.

To assess the impact of increased Shh activity on the development of dc and DP in the absence of epithelial Smo activity, we used alkaline phosphatase histochemistry. Compared to control and *Shhgfp-Cre; Smo^−/f^* skins, skin from *K14-Cre; Smo^−/f^* and *K14-Cre; Smo^f/f^* mice displayed an impressive expansion of dc and DP at all developmental stages studied (Figures 3L–3W and Table S1). These appeared as thick aggregates underlying or engulfed by follicular keratinocytes. dc/DP cells were also engulfed by epidermal keratinocytes (Figure 3T). However, these were not detected until after 2 months of age. By contrast, *K14-Cre; Shh^f/f^* and *Shh^−/−^* skin and skin grafts displayed abnormally thin DP (Figures 3N and 3X and Table S1).

Additional in situ hybridization analyses showed that the *K14-Cre; Smo^f/f^* skin displayed reduced domains of expression of markers of the hair shaft and IRS and their precursors (Figures S4A–S4J). Further, the mutant follicles were virtually devoid of the ORS markers and Shh targets *Foxe1*, *Sox9*, and *K17*, whereas the epidermal markers Keratin 1 and *Wnt7b* were ectopically expressed in the ORS, indicating its epidermal conversion (Figures S4K–S4W). However, the enlarged companion layer and differentiating hair cells expressed normal levels of *K17*. Interestingly, the IFE displayed ectopic suprabasal *K17* expression (Figure S4V–S4W), a feature that may be linked to hyperproliferation (McGowan and Coulombe, 1998). The expression patterns of *K17* in *Shh^−/−^* skin grafts were similar to those found in skin lacking epithelial Smo (Figure S4X). Analysis of cell proliferation in the *K14-Cre; Smo^f/f^* skin indicated reduced proliferation in the follicular matrix and increased epidermal and dermal proliferation (Figure S5).

**Figure 4:**
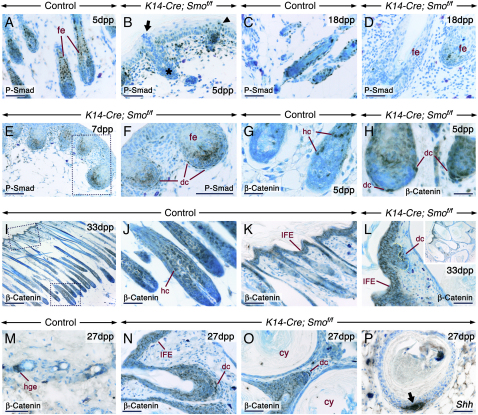
Altered Bmp and β-Catenin Signaling in the *K14-Cre; Smo^f/f^* Skin Immunostaining (dark brown) of ventral (A–F and I–L) and dorsal (G, H, and M–P) skin sections from control and mutant mice. (A–F) Phosphorylated Smad 1, 5, and 8 (P-Smad) immunostaining. (B) P-Smad-negative (arrow) and -positive (arrowhead) follicular cells at the skin surface of a mutant. The asterisk in (B) shows a gland. (D–F) Reduced numbers of P-Smad-positive cells in the mutant follicular epithelium (fe). (F) The dermal condensates (dc) associated with the mutant follicles exhibit abnormal nuclear P-Smad staining. (G–O) β-catenin immunostaining. Nuclear β-catenin staining in differentiating hair cells (hc) at anagen (G and J) and in the secondary hair germ (hge) at the telogen-to-anagen transition (M) of control follicles. (J) and (K) are high-magnification views of the indicated areas in (I). (H, L, N, and O) Increased β-catenin staining at cell-cell borders in the interfollicular epidermis (IFE) and in the follicular epithelium (L, N, and O) and decreased staining in the cyst (cy) epithelium. In addition, the mutant follicles are underlain by dc showing nuclear β-catenin staining. The dashed line in (L) marks the epithelial-mesenchymal junction in a de novo-formed follicle from the IFE. Inset in (L) is a lower magnification view. (P) Shh protein in a newly induced de novo follicle from a pre-existing follicle (arrow). Scale bars: 50 μm (A–D, F–H, J–L, and M–P), 100 μm (E), and 200 μm (I).

To assess the extent of Bmp and Wnt signaling in the *K14-Cre; Smo^f/f^* skin, we used antibodies specific to phosphorylated Smad 1, 5, and 8 (P-Smad) and to β-catenin. No major alterations in nuclear P-Smad staining were evident in mutant skin at early stages (Figure 4B). More advanced follicles displayed a severe reduction of the P-Smad-positive epithelial domain (Figures 4D–4F). In control anagen HF, nuclear β-catenin staining was patent in differentiating cells of the shaft (Figure 4J). As expected (Blanpain and Fuchs, 2006), follicles at the telogen-to-anagen transition displayed nuclear β-catenin staining in cells of the secondary hair germ (Figure 4M). Except for reduced numbers of differentiating shaft cells with nuclear β-catenin staining, no changes in staining intensities were detected in the mutant epithelium at early postnatal stages. However, from 15 dpp onward the IFE as well as the follicular epithelium displayed an increased membrane β-catenin staining at cell-cell borders, whereas the cysts showed reduced immunostaining (Figures 4L–4O). No nuclear β-catenin staining in the IFE or in the nascent de novo follicles was detected. These were consistently identified by expression of *Shh*/Shh and *Wnt10b* (Figure 4P and Figures S3L–S3P). Interestingly, dc underlying the mutant HF displayed sharp nuclear P-Smad and β-catenin staining in a subset of cells, indicating Bmp and canonical Wnt activities, respectively (Figures 4F, 4H, and 4L–4O).

Together, these data indicate that loss of follicular cells and dermal alterations are important causal factors underlying HF and IFE abnormalities in the mutants.

### Glandular Metaplasia in Ventral Skin in the Absence of Epithelial Smo Activity

Nipples and mammary glands develop in *Shh* and *Indian hedgehog* (*Ihh*) null mutants (Gallego et al., 2002) and in *K14-Cre; Smo^f/f^* females (data not shown). Further, from embryogenesis to puberty, mammary glands from *Shhgfp-Cre; R26R* mice were devoid of *lacZ*-positive cells, indicating a total absence of *Shh* expression during those stages (data not shown). Thus, it appears that Hh signaling is dispensable for early mammary gland morphogenesis.

Mammals have mammary glands exclusively in their ventrum, and many vertebrates exhibit dorsal-ventral skin differences. These patterning differences are likely to reflect the different embryonic origin of dorsal and ventral mesenchymal cells; somitic in the dorsum versus somatopleural in the ventrum (Mauger, 1972, Lowe et al., 2000).

**Figure 5:**
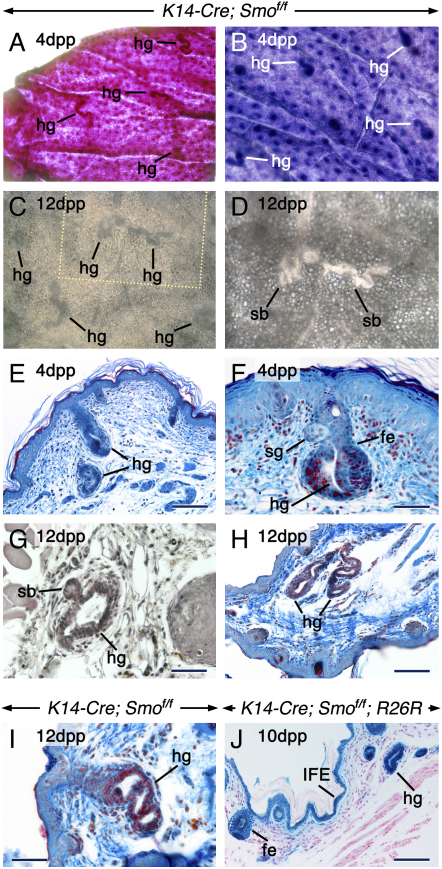
Glandular Metaplasia in Mid-Ventral Skin of *K14-Cre; Smo^f/f^* Mice (A–D) Flat-mounts of postnatal mid-ventral skin from mutants. Skin stained with neutral red (A) or hematoxylin (B). Unstained skin (C and D). The skin was photographed on its dermal (A, C, and D) or epidermal (B) side. Note the numerous heterotopic glands (hg), some of which exhibit sidebranches (sb). (D) is a high-magnification view of the area indicated in (C). (E–I) Sections from postnatal mid-ventral skin from mutants stained with Ladewig's trichrome (E, F, H, and I) or hematoxylin (G). (F) An hg that emerged from a hair follicle underneath a sebaceous gland (sg). (G) An hg with a sb. (H and I) Single hg that developed from the interfollicular epidermis (IFE). (J) Histochemistry of mid-ventral skin from a *K14-Cre; Smo^f/f^; R26R* mutant showing β-galactosidase activity in the follicular epithelum (fe), IFE, and hg. Scale bars: 50 μm (E, F, G, and I) and 100 μm (H and J).

Based on the above observations, we wondered whether the absence of epithelial Hh signaling in the HF following K14-Cre-mediated removal would be permissive to heterotopic mammary gland formation in the mid-ventrum of mutants. Indeed, we detected numerous vermiform structures in mid-ventral skin of mutants. These were never detected in the mutant dorsal skin, nor in control mid-ventral skin (Figures 5A–5D). Histology confirmed the presence of heterotopic glands (hg) and revealed that whereas some hg emerged directly from the IFE, others developed from the follicles (Figures 5E–5I). The lumina of the hg enlarged with age (Figures 5G–5I). The earliest hg were detected at 18.5 dpc in the ventral neck skin of mutants. The hg developed from ectodermal cells that underwent recombination events (Figure 5J).

**Figure 6:**
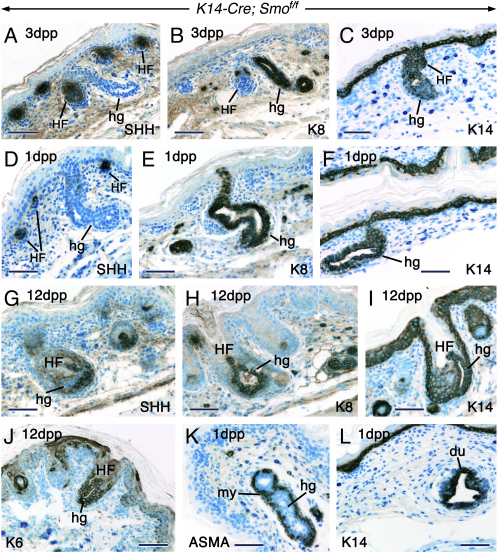
The Heterotopic Glands Emerge from the IFE, Outer Root Sheath, and Follicular Matrix Immunohistochemistry on mid-ventral skin sections from postnatal *K14-Cre; Smo^f/f^* mutants showing Shh (A, D, and G), keratin 8 (K8; [B, E, and H]), keratin 14 (K14; [C, F, I, and L]), keratin 6 (K6; [J]), and smooth muscle α-actin (ASMA; [K]) proteins. The anti-Shh antibody labels both Shh and Indian hedgehog (Ihh) proteins. (A–F) The heterotopic glands (hg) developing from hair follicles (A–C) and the hg emerging directly from the IFE (D–F) are Shh/Ihh negative and K8 positive. By contrast, the HF are Shh positive and K8 negative. Both the HF and hg are K14 positive. (G–J) The hg developing from the follicular matrix are Shh/Ihh negative and K8-, K14-, and K6 positive. (K) An ASMA-positive myoepithelium (my) around luminal cells of an hg. (L) K14-positive duct cells (du) of an orthotopic mammary gland. Scale bar: 50 μm.

In order to assess more accurately the patterns of development of the hg, their sites of emergence, as well as their identity, we used immunostaining with antibodies against Shh/Ihh, K14, K6, keratin 8 (K8), and smooth muscle α-actin (ASMA). Anti-Shh/Ihh staining showed that the mutant HF were Shh positive, whereas the hg were Shh/Ihh negative (Figures 6A, 6D, and 6G). By contrast, the hg were K8 positive, whereas the HF were K8 negative (Figures 6B, 6E, and 6H). Interestingly, K8 staining revealed cells that segregated from the matrix of some HF and formed a lumen, indicating glandular formation from the follicular matrix (Figures 6H). Luminal cells of all types of hg were K14- and K6 positive, similar to orthotopic mammary glands (Figures 6C, 6F, 6I, 6J, and 6L). In contrast to mammary glands, apocrine and eccrine sweat glands are devoid of myoepithelial cells around their ducts (Schön et al., 1999, Parmar and Cunha, 2004). Interestingly, similar to mammary glands, the hg displayed an ASMA-positive myoepithelium in their ductal portion (Figure 6K).

### Mammary Gland Metaplasia in the Ventral Skin

Whereas these analyses indicate the occurrence of glandular metaplasia in the ventral skin of *K14-Cre; Smo^f/f^* mutants, their identity remains unclear. In contrast to eccrine sweat glands, apocrine glands develop as part of the pilosebaceous unit and are under the control of sex steroid hormones, mainly androgens (Cohn, 1994). Rodents are devoid of apocrine glands in their pelage skin. They do, however, possess eccrine sweat glands in the glabrous ventral paw skin. No alterations were found in the ventral paw skin of mutants, indicating that the skin changes observed above are specific to Smo removal from haired skin (data not shown).

In the ventral skin of mutants, the occurrence of hg in association with HF points to a noneccrine nature. Further, in contrast to eccrine sweat glands (Schön et al., 1999), the ducts of both the single and follicle-associated hg were K8 positive. Finally, the dermal portion of the hg displayed wide lumina, reminiscent of those of apocrine glands which differ from the tight lumina in eccrine secretory coils. These observations suggest that the hg are not eccrine sweat glands. The hg were different from sebaceous glands morphologically (Figure 5F). Further, in contrast to hg, sebaceous glands were always K8 negative (data not shown; Kozaki et al., 2001, Schön et al., 1999).

Apocrine sweat glands do not respond to lactogenic hormones by producing milk-specific proteins, such as β-casein. To discriminate between apocrine and mammary gland metaplasia, the hg and small pieces of mid-ventral skin from mutant pups were cultured in vitro, either alone or in combination with a cleared mammary fat pad from control females. Sequential hormonal treatment generated glands that resembled mammary glands morphologically and expressed *β-casein* in the presence of a lactogenic cocktail. In vivo, the hg-expressed mammary gland genes and were devoid of *Shh*, *Ihh*, and *Ptc1* expression (See Supplemental Data, Figure S6, and Table S2).

Altogether, these findings suggest that the hg are endowed with the machinery required for mammary gland development and have the potential to differentiate into mammary glands. It is noteworthy that, in vivo, the hg lacked extensive branching morphogenesis, as they developed rather late and were located above the panniculus carnosus muscle, away from a fat pad. The mammary fat pad is known to be crucial for branching morphogenesis (Parmar and Cunha, 2004).

### Absence of Glandular Metaplasia and De Novo Folliculogenesis in the Total Absence of Shh and Absence of Major Skin Changes Following Partial Removal of Epithelial Smo Activity

Next, we wanted to determine whether glandular metaplasia as well as de novo folliculogenesis also occurred in the total absence of Shh in the skin. No morphological signs of glandular metaplasia could be detected in ventral skin from 1 dpp *K14-Cre; Shh^−/f^* or from 18.5 dpc *Shh^−/−^* mutants. Further, no skin changes were evident in 1 dpp *Shhgfp-Cre; Smo^f/f^* and *Shhgfp-Cre; Smo^−/f^* pups. These developed grossly normal HF (data not shown).

To determine whether glandular metaplasia and de novo folliculogenesis occur at later stages, we grafted ventral and dorsal skin biopsies from the above mutants into male and virgin female *nu/nu* hosts. In contrast to grafts from fetuses lacking epithelial Smo, no glandular metaplasia or de novo folliculogenesis in skin grafts from *Shh^−/−^* and *K14-Cre; Shh^f/f^* fetuses were detected. Some abnormal HF and rare glands developed in the ventral but not dorsal skin grafts from *Shhgfp-Cre; Smo^f/f^* and *Shhgfp-Cre; Smo^−/f^* fetuses (see the Supplemental Data, Figure S7, and Table S1).

Mammary glands are induced during early embryogenesis (10.5 dpc), long before induction of pelage HF (14 dpc), in an environment devoid of Hh input. This study showed that absence of epithelial Smo activity in the ventral skin induced mammary gland metaplasia. We thus wondered whether excess Shh activity as in *hK14-Shh* transgenic embryos would impair mammary gland morphogenesis. Transgenic female embryos expressing high transgene levels as indicated by their polysyndactyly displayed mammary bud anomalies ranging from absence of mammary buds, as indicated by the absence of *PTHrP*, *Tbx3*, and *Wnt10b* expression, to development of abnormally shrunken buds or branched squamous invaginations. As expected (Ellis et al., 2003, Adolphe et al., 2004), the transgenic skin developed abnormal epidermal invaginations instead of HF. Since both HF and mammary gland development were compromised in the *hK14-Shh* skin, it cannot be unequivocally concluded that excess Hh signaling is inhibitory to mammary glands. However, recent evidence suggested the requirement of Gli3-mediated repression of Hedgehog signaling for mammary gland development (Hatsell and Cowin, 2006), thus supporting our findings.

### Absence of a Distinct Follicular Stem Cell Niche in the Absence of Epithelial Smo Activity and Expansion of Mesenchymal Rapid- and Slow-Cycling Cells in Response to Increased Hh Activity

**Figure 7:**
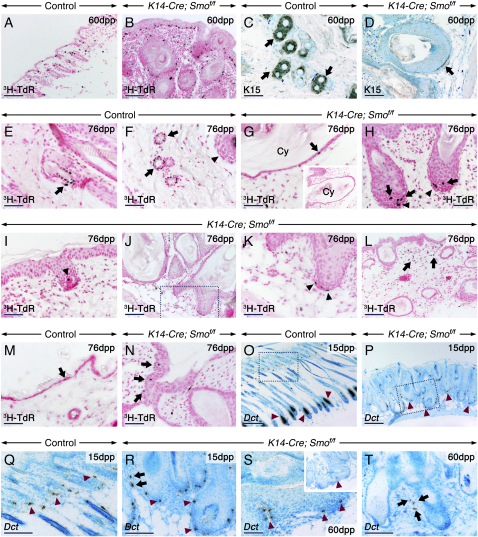
Absence of the Follicular Stem Cell Niche, Altered Number and Location of Melanocyte Stem Cells and Melanoblasts, and Increased Epidermal and Dermal Proliferation in the *K14-Cre; Smo^f/f^* Skin Skin sections from control (A, C, E, F, M, O, and Q) mutant (B, D, G–L, N, P, and R–T) mice. (A and B) Sections were processed for autoradiography 2 hr after ^3^H-TdR injection to show rapidly cycling cells (black). (C and D) Keratin 15 (K15) immunostaining (brown). Control hair follicles (HF) display K15 staining (arrows) in the bulge niche (C). (D) A mutant HF showing K15-positive cells abnormally located near the follicular matrix (arrow). (E–N) Sections processed for autoradiography 60 days after ^3^H-TdR injections to visualize label retaining cells (LRC; black). LRC representing HF stem cells (arrows) are nested in the bulge region of anagen (E) and telogen (F) control HF. Hair matrix of a pigmented HF at anagen (arrowhead in [F]). (G–L) The mutant follicles are devoid of a well-defined bulge niche with LRC. (G) A cyst wall (Cy) with a LRC (arrow). Inset in (G) is a low-magnification view. The mutant follicles contain LRC in their matrix (arrows in [H]) and in their associated dermal condensates and dermal papillae (arrowheads in [H]–[K]). (K) is a high-magnification view of the area indicated in (J). (L) Numerous stromal LRC (arrows) in the dermis of mutants. LRC (arrows) in control (M) and mutant (N) epidermises. (O–T) Bright-field images of sections hybridized with a *Dct* probe (the signal appears as black grains). Melanocyte stem cells in the bulge/sub-bulge (arrowheads in [Q]) and their amplifying progeny in the matrix (arrowheads in [O]) of control follicles. The mutant follicles show reduced numbers of *Dct*-positive cells (arrowheads in [P], [R], and [S]). Ectopic *Dct*-positive cells in the dermis of mutants (arrows in [R] and [T]). (Q) and (R) are high-magnification views of the areas indicated in (O) and (P), respectively. Inset in (S) is a low-magnification view. Scale bars: 50 μm (C–I, K, M, N, S, and T), 100 μm (A, B, J, L, Q, and R) and 200 μm (O and P).

In the *K14-Cre; Smo^f/f^* skin, the de novo development of multiple follicles, the severe hyperplasia of the IFE, the dermal hypercellularity, and increased mesenchymal Shh signaling together suggest alterations of skin homeostasis. To determine the effects of Smo ablation on skin stem cells, 10 dpp pups were injected with ^3^H-thymidine deoxyribonucleoside (^3^H-TdR) as described in Experimental Procedures. Skin biopsies from 2-month-old animals were also immunostained for Keratin 15 (K15), a marker of the bulge region (Liu et al., 2003). Pulse-labeling with ^3^H-TdR for 2 hr marked rapidly proliferating cells and highlighted the increased stromal and epidermal proliferation in skin of mutants (Figure 7B). K15 staining marked the bulge region of all control follicles (Figure 7C). In the mutants, the cysts were mostly K15 negative, whereas rare follicles contained K15-positive cells in the ORS and in the matrix (Figure 7D and data not shown). Sixty days following ^3^H-TdR injections, control anagen and telogen follicles contained ^3^H-TdR-heavily-labeled cells nested in the bulge area, indicating that they were slow-cycling label-retaining cells (LRC), whereas DP cells were devoid of labeling (Figures 7E–7F and data not shown). In the mutants, the cysts and large follicles were either devoid of labeling or contained 1–2 LRC (Figure 7G). Young de novo-formed follicles contained scattered LRC in the ORS and/or within the matrix (Figure 7H and data not shown). Interestingly, many mutant follicles displayed 1–4 stromal LRC in their dc/DP or CTS (Figures 7H–7K). Many others were underlain by dc that clearly diluted the label, indicating rapid proliferation, a feature that was not observed in control HF. Counting of dermal cells, excluding cells of the dc/DP/CTS, revealed more than a doubling in stromal LRC numbers (193.7 ± 34.0 in mutants versus 82.1 ± 24.3 in controls; p < 0.05; Student's t test) in skin of mutants (Figure 7L). A clear increase in LRC was found in the mutant IFE as well (56.2 ± 6.0 in mutants versus 32.3 ± 1.9 in controls; p < 0.01). These LRC were located at basal and suprabasal levels (Figure 7N). Due to distortion of the epithelial part of the follicles after histological processing, we were unable to make an accurate quantitative analysis to determine whether the mutant follicles developed alterations of epithelial LRC number. However, the fact that many follicles were devoid of LRC and of K15-positive cells indicates a reduced number of follicular stem cells. To determine whether some of the stromal LRC outside of dc/DP/CTS were wandering melanocyte stem cells, we examined the expression of *Dopachrome tautomerase* (*Dct*), a marker of melanocyte stem cells in the bulge region, and of their amplifying progeny in the matrix where most of them mature into melanocytes. As expected (MacKenzie et al., 1997, Nishimura et al., 2002, Slominski et al., 2005), in control HF, *Dct*-positive cells were detected in the bulge/subbulge area as well as in the upper matrix and ORS (Figures 7O and 7Q). By contrast, all mutant follicles displayed a reduced *Dct* domain in the matrix and exhibited scattered *Dct*-positive cells along the ORS. No *Dct*-positive cells were detected in the mutant IFE, whereas the dermis exhibited ectopic *Dct*-positive cells (Figures 7P and 7R–7T). These data suggest that in addition to uncharacterized rarely cycling fibroblasts, some stromal LRC in the dermis of mutants may be melanocyte stem cells. This indicates a crucial role for Shh in creating a microenvironment compatible with maintenance of the melanocyte lineage within the follicle.

## Discussion

### Shh Signaling Is Essential for Organizing and Maintaining the Integrity of the HF Epithelium

Removal of Smo activity, and consequently Hh signaling within HF epithelia, severely compromised follicular morphogenesis of all HF despite maintenance of an intact Shh responsiveness in the mesenchyme. This indicates an intrinsic requirement for Shh activity within the epithelium of the HF (Figure S8B).

In *K14-Cre; Smo^f/f^* skin, some matrix cells and IRS and shaft precursor cells were found at ectopic sites among epidermal keratinocytes. This could be a result of either or both of the following mechanisms: (1) Matrix/IRS/shaft cells were retained distally during follicular invagination. (2) These cells underwent a premature upward movement from the base of the follicle. In either case, the fate of these cells is mainly extrusion, as shown in Figure 3J. Rare dc/DP clusters were also found within the IFE. These could comprise dc/DP cells that remained trapped in the epidermis after migrating outwards with follicular keratinocytes. The exact causes of these anomalies are unclear. Interestingly, apolarized *Shh* expression, altered HF morphogenesis, and DP cell ectopia in the epidermis were recently described in skin lacking Dicer (Andl et al., 2006, Yi et al., 2006).

In *K14-Cre; Smo^f/f^* skin, although some rudimentary IRS and shaft differentiated, the abnormal HF totally lacked the typical laminar architecture of the IRS and shaft. Further, over time, many follicles were reduced to cysts. These changes are likely to be secondary to an abnormal loss of IRS and shaft precursor cells, combined with lowered production of progenitors due to altered cell proliferation and loss of proliferating cells (Figure S8B).

Our findings shed light onto the cellular events leading to abnormal IRS/shaft development in the mutants and point to an essential function for Shh in maintaining HF integrity necessary for the generation and organization of IRS and shaft cells and their precursors.

In contrast to skin grafts from *K14-Cre; Smo^f/f^* mice, grafts from *Shhgfp-Cre; Smo^f/f^* mice developed grossly normal fur, aside from rare abnormal ventral follicles. Our cell fate analysis indicated that in contrast to the *K14* promoter, the *Shh* regulatory sequences drove Cre activity in some but not all ORS precursors. These data indicate that Shh responsiveness within the developing ORS is necessary for normal hair morphogenesis. Transcripts of the ORS markers and Shh target genes *Foxe1*, *Sox9*, and *K17* were virtually absent, whereas *Wnt7b* and Keratin 1 were present ectopically in the ORS of *K14-Cre; Smo^f/f^* follicles, indicating conversion of the follicular ORS into epidermis (Figure S8B). Thus, Shh is also crucial for specifying and/or maintaining the identity of the ORS.

### Shh Is Crucial for Development of the Follicular Stem Cell Niche and for Stem Cell Homeostasis

The general shape and organization of the follicles were similar in skin grafts from *Shh^−/−^* (St-Jacques et al., 1998, Chiang et al., 1999; this study), *K14-Cre; Shh^f/f^*, and *K14-Cre; Smo^f/f^* (this study) mutants. However, the Shh*-*deleted skin grafts did not display de novo folliculogenesis, and the dc/DP were hypoplastic. Together with the known function of DP cells in inducing HF (Jahoda and Reynolds, 1993), and the fact that the de novo folliculogenesis in the *K14-Cre; Smo^f/f^* and *K14-Cre; Smo^−/f^* mutants was confined to haired skin, these results point to the involvement of a Shh-dependent mesenchymal input required to induce de novo HF.

In the absence of epithelial Smo function, abnormally increased expression levels of *Ptc1* and *Gli1* in both dc/DP and dermis underlying the IFE indicate an increased orthotopic and ectopic Shh activity, respectively (Figure S8D). Further, increased proliferation in the dermis and CTS, the increased presence of stromal LRC in dc/DP/CTS, and the presence of dc/DP cells that diluted the ^3^H-TdR label indicate an expansion of the HF-associated dermal population. This was further clearly shown by an impressive expansion, both orthotopically and ectopically, of alkaline phosphatase-positive cell aggregates typifying dc/DP. These changes are likely to be secondary to pathway activation via an augmented Shh protein availability following epithelial Ptc1 downregulation, a condition that was not met in the absence of Shh.

Shh is a bona fide mitogen operating in several embryonic and adult organs, and pathway activation may lead to neoplasia (McMahon et al., 2003). Although seemingly paradoxical, the de novo formation of follicles is consistent with the fact that Shh is not necessary for HF induction (St-Jacques et al., 1998, Chiang et al., 1999). More intriguing, however, was the development of as many as 5–7 follicles from numerous pre-existing follicles in 2- to 3-month-old *K14-Cre; Smo^f/f^* mutants. Knowing that in the mouse, hair cycles approximately every 4 weeks, one would have expected development of one or two new follicles in 2- and 3-month-old mice, respectively. Moreover, the first de novo follicles were detected at 7 dpp, well ahead of the first hair cycle, whereas secondary de novo follicles emerged from pre-existing ones. Our ^3^H-TdR labeling and K15 staining showed that, in contrast to control follicles which displayed a well-defined bulge niche, the mutant follicles were either devoid of or contained a few scattered LRC and K15-positive cells along the ORS as well as within their matrix, implying the absence of a protective bulge niche for putative stem cells. This was also shown by the total absence of expression of the bulge marker *Sox9*.

At anagen, quiescent stem cells are situated at a distance from DP cells. By showing that this situation was not maintained in follicles lacking epithelial Smo activity, we were able to solve the above conundrum. Thus, pathway inactivation leading to absence of the stem cell niche and constant solicitations by an expanded population of dermal cells likely caused quiescent epithelial stem cells to enter the transient-amplifying pool precociously and generate multiple follicles from pre-existing ones.

### Relationship between Shh and Wnt Signaling

The Wnt/β-catenin pathway is a key player in epidermal cell fate specification and homeostasis. Thus, transgenic expression of a stabilized form of β-catenin prompts hair growth or induces HF tumors and de novo HF formation. Conversely, ablation of Wnt responses abrogates HF formation and promotes epidermal and sebaceous cell fates (reviewed in Blanpain and Fuchs, 2006).

In the *K14-Cre; Smo^f/f^* skin, the conversion of the ORS into epidermis, the occurrence of de novo HF formation, and cyst development (Figure S8B) recapitulate some of the changes described following both β-catenin gain- and loss-of-function (Blanpain and Fuchs, 2006). However, no epithelial nuclear β-catenin staining was evident in the IFE and during early stages of de novo folliculogenesis. Instead, a prominent increase in β-catenin staining at cell-cell borders was noted at late postnatal stages. Whether this reflects decreased β-catenin activity needs further analyses. However, this could be a possibility, at least after induction of ectopic HF has taken place, in light of recent evidence suggesting Shh-dependent β-catenin stabilization in HF (Mill et al., 2005). In contrast to the situation following β-catenin activation (Blanpain and Fuchs, 2006), in the *K14-Cre; Smo^f/f^* mutants the first de novo-generated follicles appeared before initiation of the first hair cycle. Furthermore, follicles emerged from any part of the ORS and were not restricted to its permanent segment. Our data suggested the occurrence of Shh-dependent mesenchymal signals in the generation of de novo follicles. Interestingly, Shh pathway activation consistently takes place following β-catenin activation, and Shh signaling seems to be required for β-catenin-induced de novo HF formation (Silva-Vargas et al., 2005, Blanpain and Fuchs, 2006).

Altogether, these data suggest that the Shh and Wnt pathways are interwoven and play a determinant role in epidermal cell fate determination.

Recent work suggested the involvement of DP factors that may act in conjunction with Wnt/β-catenin to induce HF stem cell activation (Blanpain and Fuchs, 2006). Interestingly, in the *K14-Cre; Smo^f/f^* skin, a sharp nuclear β-catenin staining was detected in a subset of dc/DP cells, and this trend seemed to be a rule rather than an exception. However, the functional significance of this is unclear at present.

### Shh Controls Follicular and Mammary Gland Cell Fate Allocation

Epithelial Smo ablation generated HF in which the ORS converted into epidermis. Further, in the absence of epithelial Smo, ventral but not dorsal skin displayed glandular metaplasia (Figures S8E and S8F), where follicular keratinocytes were diverted totally or partially into glands endowed with the potential to differentiate into mammary glands. The ventral and dorsal follicles displayed similar molecular changes. Thus, it seems that absence of epithelial Smo activity is necessary but not sufficient to elicit glandular metaplasia, and that the dorsal-ventral differences in the phenotypic outcome are solely due to differences in mesenchymal influences.

Total ablation of Shh signaling, as in Shh null skin and skin grafts, did not induce glandular metaplasia, probably due to insufficient number of mesenchymal cells necessary to elicit epithelial cell fate change into glands. Partial loss of Shh signaling, as in *Shhgfp-Cre; Smo^f/f^* skin grafts, was permissive to glandular metaplasia only at low frequency. This is likely due to the rarity of HF that underwent abnormal development. Thus, it seems that epithelial-specific ablation of Smo permits sufficient mesenchymal development to allow the visualization of an unanticipated role for Shh in permitting hair follicle development by suppressing inappropriate mammary gland fates.

In the *K14-Cre; Smo^f/f^* ventral skin, the earliest molecular changes preceding glandular metaplasia include K8 staining and absence of *Shh* expression in glandular cells. Since only a small population of matrix cells expresses *Shh* in HF, it cannot be stated that in the follicle/gland hybrids the glands emerged from cells that had lost *Shh* expression. However, in glands that emerged directly from the IFE, it was clear that follicular keratinocytes that adopted a glandular fate had lost *Shh* expression. It has been shown that loss of *Shh* is necessary but not sufficient for conversion of stomach and gut endoderm into pancreas or for induction of proventricular glands in the chick stomach. These events appear to require additional mesenchymal cues (Kim and Melton, 1998, Fukuda et al., 2003). Those data would be consistent with ours, as no loss of *Shh* expression was found in the dorsal mutant HF, thus indicating the existence of additional ventral mesenchymal influences causing glandular metaplasia.

The hg emerged at different levels along the ORS as well as from the matrix of the mutant HF. It is likely that glandular metaplasia took place in uncommitted stem cells that were found at those locations in the follicles as indicated by ^3^H-TdR labeling and K15 staining. Other hg emerged directly from the epidermis, indicating that they developed at the expense of HF.

Human pathology provides many examples of metaplasia, which is considered as a prelude to neoplasia, and cases of mammary tissue outside the “milk line” have been described in humans (Grossi, 2000). Insights from genetic manipulation suggest that metaplasia arises from the change in expression levels of key developmental genes (Tosh and Slack, 2002). By showing that the sole loss of epithelial Smo activity caused glandular metaplasia, we can conclude that Shh operates at the top of a molecular hierarchy that specifies follicular versus glandular cell fate decisions in response to specific mesenchymal signals.

The mammary gland emerged late during evolution and adopted a number of signaling pathways operating in other ectodermal organs, such as hairs (Oftedal, 2002). Identifying the mesenchymal signals that prevent *Shh* and *Ihh* expression during mammary gland induction will shed light into its evolutionary origin, which is not well understood.

## Experimental Procedures

### Generation of Mutants

Generation of the different mutants was as described in the Supplemental Experimental Procedures.

### Histology, Histochemistry, Immunohistochemistry, and In Situ Hybridization

Skin specimens from controls and mutants were fixed either in 4% paraformaldehyde or in Sainte Marie's fixative (Gritli-Linde et al., 2001) and processed for paraffin embedding. For histology, sections were stained with Ladewig's trichrome or with Alcian Blue-van Gieson stains.

Alkaline phosphatase activity was visualized on cryostat sections from formalin-fixed skin using a substrate kit from Sigma.

Immunostaining using enzyme substrates that generate a brown or magenta precipitate was performed essentially as described previously (Gritli-Linde et al., 2001). The antibodies and dilutions used are listed in the Supplemental Experimental Procedures.

The probes used for in situ hybridization are listed in the Supplemental Experimental Procedures.

### Localization of Rapid- and Slow-Cycling Cells

For detection of rapidly proliferating cells, animals received a single intraperitoneal injection (ip) of 5 μCi/g body weight (bw) [methyl-^3^H] thymidine deoxyribonucleoside (^3^H-TdR; specific activity 25 Ci/mmol; Amersham Bioscience), and skin biopsies were taken after 2 hr. For detection of slow-cycling, label-retaining cells (LRC), 10 dpp pups were injected ip with 5 μCi/g bw ^3^H-TdR for 6 consecutive days, and skin biopsies were harvested 54–60 days after the last injection. Paraffin sections were processed for autoradiography and counterstained with Nuclear Fast Red. LRC were also identified by autoradiography in skin biopsies from ^3^H-TdR-injected *Shhgfp-Cre; R26R* mice after β-galactosidase staining. To determine the quantitative changes of LRC in the interfollicular epidermis and in the dermis (excluding the dermal condensates/DP/CTS) in mutant versus controls, the number of LRC was counted in two-hundred ×40 microscope objective fields per specimen (six animals per group). For counting, the epidermis was positioned along the diameter of the circular field. Statistical analysis was made by using Student's t test.

## Supplemental Data

Document S1. Supplemental Results, Supplemental Experimental Procedures, Two Tables, and Eight Figures
