# Supplementary material for: Abnormal Hair Development and Apparent Follicular Transformation to Mammary Gland in the Absence of Hedgehog Signaling
Source: Dev Cell. 2007 Jan;12(1):99–112. doi: 10.1016/j.devcel.2006.12.006 (PMC1885956; doi:10.1016/j.devcel.2006.12.006)
Supplement: Document S1. Supplemental Results, Supplemental Experimental Procedures, Two Tables, and Eight Figures [file mmc1.pdf]

Developmental Cell *12*

Supplemental Data

Abnormal Hair Development and Apparent Follicular Transformation to Mammary Gland in the Absence of Hedgehog Signaling

Amel Gritli-Linde, Kristina Hallberg, Brian D. Harfe, Azadeh Reyahi, Marie Kannius-Janson, Jeanette Nilsson, Martyn T. Cobourne, Paul T. Sharpe, Andrew P. McMahon, and Anders Linde

Supplemental Results

β-Galactosidase Histochemistry Reveals Differences between K14-Cre- and Shhgfp-Cre-Mediated Recombination Events at the *R26R* Reporter Locus

To visualise cells that underwent Cre-mediated recombination events at the *R26R* locus as well as their progeny, *K14-Cre; R26R* and *Shhgfp-Cre; R26R* skins were processed for β-galactosidase histochemistry. Both during embryogenesis and postnatally, the *K14-Cre; R26R* skin displayed robust β-galactosidase staining in all cells of the epidermis and pilosebaceous unit (Figures S1A-D). However, the neural crest-derived melanocytes and their progenitors (Mayer, 1973), which lack epithelial specialization and are devoid of K14 expression (Peters et al., 2002), are not expected to undergo recombination events and thus, remain Shh responsive. These cells should thus be *lacZ*-negative in the *K14-Cre; R26R* skin, but are likely masked by the robust staining of adjacent keratinocytes. *LacZ*-negative cells could be detected in a domain encompassing that of melanocytes in 4μm thin sections (Figures S1E-F).

In contrast to *K14-Cre; R26R* skin, β-galactosidase-stained skin sections from *Shhgfp-Cre; R26R* embryos and postnatal animals (Figures S1G-R) revealed a subset of *lacZ*-negative follicular cells from early stages onwards. Specifically, some cells of the hair

germ were negative, and during anagen the *lacZ*-negative domain became confined to a subset of ORS cells, whereas sebaceous glands were either *lacZ*-positive or negative. During telogen of the first and second HF cycles, the bulge (stem cell region) contained both *lacZ*-positive and *lacZ*-negative cells (Figure S1L). This was supported by ^{3}H-thymidine deoxyribonucleoside (^{3}H-TdR) autoradiography (Figures S1M-R). Only a subset of bulge *lacZ*-negative cells may represent melanocytes stem cells as HF at telogen contain only 1-3 *Dopachrome tautomerase* (*Dct*)-positive cells (data not shown; Nishimura et al., 2002). In contrast to a recent report (Levy et al., 2005**)**, these data indicate that from early stages of HF formation there exists a subpopulation of follicular cells that are not the progeny of cells that have expressed *Shh*. Alternatively, at the placodal stage, some cells might have escaped Shhgfp-Cre-mediated recombination. The exact reasons for the discrepancies between our data and those of Levy et al. (2005) are not known. β-galactosidase staining patterns in our specimens could be due to cell-to-cell variations in the levels of *lacZ* expression. However, our skin graft analyses clearly showed that in contrast to *K14-Cre; Smo$^{f/f}$* and *K14-Cre; Smo$^{-/f}$* follicles which displayed severe defects of the ORS and bulge area, the ORS and bulge were unaltered in the majority of the *Shhgfp-Cre; Smo$^{f/f}$* and *Shhgfp-Cre; Smo$^{-/f}$* follicles indicating incomplete Shhgfp-Cre mediated recombination in the early embryonic precursors of these structures, thus ruling out the occurrence of variations in *lacZ* expression levels.

Transcriptional and Proliferation Alterations in the Absence of Epithelial Smo Activity

To determine the molecular basis of aberrant skin development in *K14-Cre; Smo$^{f/f}$* and *K14-Cre; Smo$^{-/f}$* mice, we analysed the expression patterns of genes encoding factors controlling HF morphogenesis and differentiation, including *Bmp2*, *Bmp4*, *Hoxc13*, *Msx1*, *Msx2*, *Wnt5a* and *Epiprofin* (Godwin and Capecci, 1998; Kulessa et al., 2000; Nakamura et al., 2000; Satokata et al., 2000; Reddy et al., 2001; Kobielak et al., 2003; Andl et al., 2003; Yuhki et al., 2004). Additional analyses included the epidermal markers *Wnt7b* (Reddy et al., 2001) and Keratin 1 as well as the ORS markers and Shh targets in HF and follicular tumors, including *Keratin 17*, *Foxe1* and

Sox9 (McGowan and Coulombe, 1998; Mill et al., 2003; Oro and Higgins, 2003; Callahan et al., 2004; Brancaccio et al., 2004; Bianchi et al., 2005; Hutchin et al., 2005; Vidal et al., 2005; Svärd et al., 2006). Cell proliferation was assessed using ^{3}H-TdR labeling, anti-Ki 67 immunostaining and in situ hybridization with a *Cyclin D1* probe. (Data are summarized in Table S2; see also Figures S4 and S5).

The Heterotopic Glands (hg) Differentiate into Mammary Glands In Vitro and Express Mammary Gland Markers

After *in vitro* culture in the presence of 17β-estradiol and progesterone, the hg and mid-ventral skin explants generated well developed glandular ductal structures (Figures S6A-B). In the presence of a lactogenic cocktail (dexamethasone and prolactin), the distal portions of the ductal structures displayed alveoli filled with secretory products (Figure S6D). No glandular formations were obtained from mutant and control dorsal skin, nor from control ventral skin (Figures S6C and S6F and data not shown). Cultures of recombinants made of hg and cleared mammary fat pads displayed increased ductal and alveolar development after a permissive sequential hormonal treatment (Figure S6E). The hg expressed *β-casein* after treatment with the lactogenic cocktail (Figures S6G-H), but not in its absence (data not shown).

Both the hg associated with the mutant follicles and the hg that emerged from the IFE expressed genes that are normally expressed during mammary gland development, including *Barx2*, *Sox9*, *Tbx3* (Krasner et al., 2000; Pask et al., 2002; Davenport et al., 2003) and *Wnt7b* (this study), and the expression levels were similar to those of control orthotopic mammary glands. Finally, similar to orthotopic mammary glands, the hg were devoid of *Shh, Ptc1* and *Ihh* (Figures S6J-M and data not shown).

Absence of Glandular Metaplasia and De Novo Folliculogenesis in the Total Absence of Shh and Absence of Major Skin Changes Following Partial Removal of Epithelial Smo Activity

Four to 6 weeks post-grafting, dorsal and ventral grafts from control fetuses produced normal furs with the ventral ones being less dense than the dorsal, consistent with

dorsal-ventral differences in pelage hair (Figures S7A-B and S7I-J). Dorsal and ventral skin grafts from *Shh*$^{-/-}$, *K14-Cre; Shh*$^{-/f}$ or *K14-Cre; Shh*$^{f/f}$ fetuses were devoid of hair, and the ventral grafts were essentially devoid of hair follicles (Figures S7C-D and Figures S7K-L, and data not shown). No glandular metaplasia was evident in the ventral grafts analysed (n=4). In addition, there was no evidence of de novo follicular formation in dorsal skin grafts from the above mutants. Dorsal skin grafts from *Shhgfp-Cre; Smo*$^{-/f}$ (data not shown) and *Shhgfp-Cre; Smo*$^{f/f}$ fetuses produced a dense fur. However, ventral skin grafts produced less dense furs than the control ventral grafts (Figures S7E-F). While dorsal HF were normal (Figure S7M-N), some abnormal follicles developed in ventral skin grafts (Figure S7O). Furthermore, in the latter, one graft displayed a hg arising directly from the epidermis (Figure S7P). Dorsal and ventral skin grafts from *K14-Cre; Smo*$^{-/f}$ (data not shown) and *K14-Cre; Smo*$^{f/f}$ fetuses were completely devoid of hair (Figures S7G-H). Ventral grafts displayed hg, many of which contained pigmented melanocytes (Figure S7U). In addition, numerous pigmented melanocytes were found in the dermis. Some of the melanocytes colonized the host albino follicles in post-healing fusion areas between donor and host sites and produced pigmented hairs with twisted hair shaft, a characteristic of the *nu/nu* HF (Figures S7Q-T). Finally, numerous de novo-generated follicles were found in both ventral and dorsal skin grafts (Figures S7T and data not shown).

Thus, in the total absence of Shh as in *Shh*$^{-/-}$ and *K14-Cre; Shh*$^{-/f}$ and *K14-Cre; Shh*$^{f/f}$ grafts, glandular metaplasia failed to develop, whereas partial epithelial loss of Shh responsiveness, as in *Shhgfp-Cre; Smo*$^{f/f}$ and *Shhgfp-Cre; Smo*$^{-/f}$ grafts was permissive to glandular metaplasia, although at a much lower frequency than in *K14-Cre; Smo*$^{f/f}$ and *K14-Cre; Smo*$^{-/f}$-mutants. In addition, both the total and partial absence of Shh signaling were not permissive for de novo folliculogenesis (The data are summarized in Table S1).

Supplemental Experimental Procedures

Generation of Mutant and Transgenic Animals and β-Galactosidase Staining

Transgenic mice expressing Cre recombinase under the control of Shh regulatory sequences (*Shhgfp-Cre/+*) or under the regulation of the enhancer element of *cytokeratin 14* (*K14-Cre/+*) as well as the *R26R* conditional reporter mice have been described previously (Soriano, 1999; Dassule et al., 2000; Harfe et al., 2004). The *Smo* and *Shh* conditional alleles (floxed; [f]) generated by flanking the first and second essential exons, respectively, with *loxP* sites were as described (Dassule et al., 2000; Long et al., 2001; Zhang et al., 2001; Lewis et al., 2004). The *Smo* and *Shh* null alleles were as described (St-Jacques et al., 1998; Zhang et al., 2001).

Males heterozygous for both the *K14-Cre* transgene and the *Smo* null or *Smo* floxed alleles were mated with $Smo^{f/f}$ females to generate mutants (*K14-Cre;* $Smo^{-/f}$ or *K14-Cre;* $Smo^{f/f}$). Mutant embryos and newborns lacking Shh protein within the ectoderm (*K14-Cre;* $Shh^{f/f}$ and *K14-Cre;* $Shh^{-/f}$) were generated by crossing males heterozygous for both the *K14-Cre* transgene and the Shh floxed or *Shh* null alleles (*K14-Cre;* $Shh^{+/f}$ or *K14-Cre;* $Shh^{+/-}$) with $Shh^{f/f}$ females. $Shh^{-/-}$ mutants (St-jacques et al., 1998) were generated by crossing $Shh^{+/-}$ males with $Shh^{+/-}$ females. Mice lacking Smo activity in cells that express or have expressed *Shh* were generated by crossing double heterozygous males (*Shhgfp-Cre;* $Smo^{+/f}$ or *Shhgfp-Cre;* $Smo^{+/-}$) with $Smo^{f/f}$ females. Transgenic embryos overexpressing Shh under the control of the human K14 promoter (*hK14-Shh*) were generated as previously described (Adolphe et al., 2004).

The different lines were genotyped by PCR as described previously (Soriano, 1999; Dassule et al., 2000; Long et al., 2001; Zhang et al., 2001; Adolphe et al., 2004; Harfe et al., 2004; Lewis et al., 2004).

The spatiotemporal patterns of Cre-mediated recombination events were determined by β-galactosidase staining of skin specimens from *K14-Cre; R26R*, *Shhgfp-Cre; R26R* as well as from *K14-Cre;* $Smo^{f/f}$*; R26R* mice at different developmental stages.

For β-galactosidase staining, tissues were processed essentially as described earlier (Vaziri Sani et al., 2005). After fixation, skin from the above transgenic lines was stained for β–galactosidase, embedded in paraffin, sectioned and counterstained with Nuclear Fast Red.

Antibodies and Riboprobes

Anti-Shh/Ihh (diluted 1:2000) was as described (Bumcrot et al., 1995; Gritli-Linde et al., 2001). The Troma-1 rat monoclonal antibody (1:2000) developed by P. Brulet and R. Kemler was obtained from the DSHB developed under the auspices of the NICHD and maintained by the University of Iowa, Department of Biological Sciences, Iowa City, IA, USA. Other antibodies were as follows: mouse anti-cytokeratin 14 (1:400; clone LL002, YLEM, Rome, Italy); rat anti-E-cadherin (1:600), mouse anti-β-catenin (1:400, clone CAT-5H10) and rabbit anti-ZO-1 (1:1000) were from Zymed Laboratories, South San Francisco, CA, USA; rabbit monoclonal anti-cleaved caspase-3 (1:1000; Asp175) and rabbit anti-phospho-Smad1, 5, 8 (1:500) were from Cell Signaling Technology, Beverly, MA, USA; rabbit anti-keratin1 (1: 4000), rabbit anti-keratin 6 (1:8000) and chicken anti-keratin 15 (1: 10,000) were from Covance, Berkley, CA, USA; mouse anti-smooth muscle α-actin (1: 2000; clone 1A4) was from Sigma, St Louis, MO, USA; goat anti-syndecan-1 (1:400) was from Santa Cruz Biotechnology, Santa Cruz, CA, USA; and rat anti-Ki 67 (1: 500) was from DAKO, Glostrup, Denmark.

The following probes were used for radioactive in situ hybridization: *Barx2*, *Bmp2*, *Bmp4*, *β-casein*, *Cyclin D1*, *Dct* (also known as *Trp2* encodes dopachrome tautomerase), *Epiprofin*, *Foxe1*, *Gli1-3*, *Hoxc13*, *Ihh*, *K17*, *Msx1*, *Msx2*, *Ptc1*, *PTHrP*, *Shh*, *Smo*, *Sox9*, *Tbx3*, *Wnt5a*, *Wnt7b*, and *Wnt10b*. In situ hybridization was performed on sections of both dorsal and ventral skin specimens from control and mutant unpigmented animals and processed on the same histological slide. Bright-field images were included in some panels to avoid artefacts generated by keratin deposits and tissue distorsion which hamper signal visualization in dark-field images. Hybridization signals appear as black or bright grains in bright-field and dark-field

images, respectively. In some panels, silver grains were pseudo-colored red using PhotoShop.

In Vitro Culture of Skin Biopsies

All culture media and supplements were purchased from Invitrogen, hormones from Sigma. Small pieces of dorsal and mid-ventral skin fragments (excluding mammary glands/ducts) were dissected out from 3-5 days post-partum (dpp) control and *K14-cre; Smo*$^{c/c}$ mutant animals. Single or follicle-associated heterotopic glands (see Results) that developed in the mid-ventrum of mutants were also easily dissected out as single explants. The explants were either cultured alone or mixed with cleared mammary fat pads (De Ome et al., 1959) from 2.5 week-old female mice. The explants were grown on 0.45 µm pore size Millipore filters supported by metal grids in a humidified atmostphere of 5% CO_2 in air at 37°C and in the presence of α-MEM medium, 2 mM Glutamax, 10% fetal bovine serum and antibiotics. After 24 hours, the medium was changed into a serum-free basal medium, a mix (1:1) of Dulbecco's MEM medium and M-199 supplemented with 2 mM Glutamax, antibiotics, 2 µl/ml bovine pituitary extract, 40 µg/ml bovine transferrin, 5 µg/ml insulin, 150 µg/ml ascorbic acid, 0.2% BSA, 12 ng/ml EGF. After 1 or 2 weeks, the explants were cultured for an additional 2 or 3 weeks in the above basal medium supplemented with 1 ng/ml 17-β-estradiol and 1 µg/ml progesterone. After estrogen/progesterone treatments, some explants were cultured for an additional 1 or 2 weeks in a medium containing the lactogenic hormones prolactin (5 µg/ml) and dexamethasone (1 µM) and devoid of estrogen/progesterone. Cultured explants were processed for paraffin embedding, histology and in situ hybridization.

Skin Grafting

Full-thickness midventral and dorsal skin biopsies taken from 18.5 days post-coitum (dpc) embryos were grafted onto similar size (≈ 8 x 6-8 mm) back skin recipient sites in anesthesized male or female *nu/nu* immunodeficient mice. The grafts were protected by surgical tape and bandage which were removed one week post-surgery.

Supplemental References

Adolphe, C., Narang, M., Ellis, T., Wicking, C., Kaur, P., and Wainwright, B. J. (2004). An in vivo comparative study of Sonic, Desert and Indian Hedgehog reveals that Hedgehog pathway activity regulates epidermal stem cell homeostasis. Development *131*, 5009-5019.

Andl, T., Ahn, K., Kairo, A., Chu, E.Y., Wine-Lee, L., Reddy, S.T., Croft, N.J., Cebra-Thomas, J.A., Metzger, D., Chambon, P., Lyons, K.M., Mishina, Y., Seykora, J.T., Crenshaw, E.B.III., and Millar, S.E. (2004). Epithelial Bmpr1a regulates differentiation and proliferation in postnatal hair follicles and is essential for tooth development. Development *131*, 2257-2268.

Bianchi, N., DePianto, D., McGowan, K., Gu, C., and Coulombe, P.A. (2005). Exploiting the Keratin 17 gene promoter to visualize live cells in epithelial appendages of mice. Mol. Cell. Biol. *25* 7249-7259.

Brancaccio, A., Minichiello, A., Grachtchouk, M., Antonini, D., Sheng, H., Parlato, R., Dathan., N., Dlugosz, A., and Missero, C. (2004). Requirement of the forkhead gene *Foxe1*, a target of sonic Hedgehog signaling, in hair follicle morphogenesis. Hum Mol. Genet. *13*, 2595-2606.

Bumcrot, D.A., Takada, R., and McMahon, A.P. (1995). Proteolytic processing yields two secreted forms of sonic hedgehog. Mol. Cell. Biol. *15*, 2294-2303.

Callahan, C.A., Ofstad, T., Horng, L., Wang, J.K., Zhen, H.H., Coulombe, P.A., and Oro, A.E. (2004). MIM/BEG4, a Sonic hedgehog-responsive gene that potentiates Gli-dependent transcription. Genes Dev. *18*, 2724-2729.

De Ome, K.B., Faulkin, L.J., and Bern, H.A. (1959). Development of mammary tumors from hyperplastic alveolar nodules transplanted into gland-free mammary fat pads of female C3H mice. Cancer Res. *19*, 515-520.

Dassule, H.R., Lewis, P., Bei, M., Maas, R., and McMahon, A.P., 2000. Sonic hedgehog regulates growth and morphogenesis of the tooth. Development *127*, 4775-4785.

Davenport, T.G., Jerome-Majewska, L.A., and Papaioannou, V.E. (2003). Mammary gland, limb and yolk sac defects in mice lacking *Tbx3*, the gene mutated in ulnar mammary syndrome. Development *130*, 2263-2273.

Godwin, A.R., and Capecci, M.R. (1998). *Hoxc13* mutant mice lack external hair. Genes Dev. *12*, 11-20.

Gritli-Linde, A., Lewis, P., McMahon, A.P., and Linde, A. (2001). The whereabouts of a morphogen: Direct evidence for short- and long-range activity of Hedgehog signaling peptides. Dev. Biol. *236*, 364-386.

Harfe, B.D., Schertz, P.J., Nissim, S., Tian, H., McMahon, A.P., and Tabin, C.J., 2004. Evidence for an expansion-based temporal Shh gradient in specifying vertebrate digit identities. Cell *118*, 517-528.

Hatsell, S.J., and Cowin, P. (2006). Gli3-mediated repression of Hedgehog targets is required for normal mammary development. Development *133*, 3661-3670.

Hutchin, M.E., Kariapper, M.S.T., Grachtchouk, M., Wang, A., Wei, L., Cummings, D., Liu, J., Michael, L.E., Glick, A., and Dlugosz, A. (2005). Sustained hedgehog signaling is required for basal cell carcinoma proliferation and survival: conditional skin tumorigenesis recapitulates the hair growth cycle. Genes Dev. *19*, 214-223.

Kobielak, K., Pasolli, A.H., Alonso, L., Polak, L., and Fuchs E. (2003). Defining BMP function in the hair follicle by conditional ablation of BMP receptor IA. J. Cell Biol. *163*, 609-623.

Krasner, A., Wallace, L., Thiagalingam, A., Jones, C., Lengauer, C., Minahan, L., Ma, Y., Kalikin, L., Feinberg, A.P., Jabs, E.W., Tunnacliffe, A., Baylin, S.B., Ball., D.W., and Nelkin, B.D. (2000). Cloning and chromosomal localization of the human *BARX2* homeobox protein gene. Gene *250*, 171-180.

Kulessa, H., Turk, G., and Hogan, B.L.M. (2000). Inhibition of Bmp signaling affects growth and differentiation in the anagen hair follicle. EMBO J. *19*, 6664-6674.

Lewis, P.M., Gritli-Linde, A., Smeyne, R. Kottmann, A., and McMahon, A.P. (2004). Sonic hedgehog signaling is required for expansion and patterning of the mouse cerebellum. Dev. Biol. *270*, 393-410.

Long, F., Zhang, X.M., Karp, S., Yang, Y., and McMahon A.P**.** (2001). Genetic manipulation of hedgehog signaling in the endochondral skeleton reveals a direct role in the regulation of chondrocyte proliferation. Development *128*, 5099-5108.
McGowan, K.M., and Coulombe, P. (1998). Onset of keratin expression coincides with the definition of major epithelial lineages during skin development. J. Cell. Biol. 143, 469-486.

Mill, P., Mo, R; Fu, H., Grachtchouk, M., Kim, P.C.W., Dlugosz, A.A., and Hui, C-C. (2003). Sonic hedgehog-dependent activation of Gli2 is essential for embryonic hair follicle development. Genes Dev. *17*, 282-294.

Nakamura, T., Unda, F., de-Vega, S., Vilaxa, A., Fukumoto, S., Yamada, K.M., and Yamada Y. (2004). The krüppel-like factor Epiprofin is expressed by epithelium of developing teeth, hair follicles, and limb buds and promotes cell proliferation. J. Biol. Chem. *279*, 626-634.

Oro, A.E., and Higgins, K. (2003). Hair cycle regulation of Hedgehog signal reception. Dev. Biol. *255*, 238-248.

Pask, A.J., Harry, J.L., Marshall Graves, J.A., Waugh O'Neill, R.J., Layfield, S.L., Shaw, G., and Renfree, M.B. (2002). Sox9 has both conserved and novel roles in marsupial sexual differentiation. Genesis *33*, 131-139.

Reddy, S., Andl, T., Bagasra, A., Lu, M.M., Epstein, D.J., Morrisey E.E., and Millar, SE (2001). Characterization of Wnt gene expression in developing and postnatal hair follicles and identification of Wnt5a as a target of Sonic hedgehog in hair morphogenesis. Mech. Dev. *107*, 69-82.

Satokata, I., Ma, L., Ohshima, H., Bei, M., Woo, I., Nishizawa, K., Maeda, T., Takano, Y., Uchiyama, M., Heaney, S., Peters, H., Tang, Z, Maxson, R., and Maas, R. (2000). Msx2 deficiency in mice causes pleiotropic defects in bone growth and ectodermal organ formation. Nature Genet. *24*, 391-395.

Soriano, P., 1999. Generalized lacZ expression with the ROSA26 Cre reporter strain. Nat. Genet. *21*, 70-71.

St.-Jacques, B., Dassule, H., Karavanova, I., Botchkarev, V., Li, J., Danielian, P., McMahon, J., Lewis, P., Paus, R., and McMahon, A.P. (1998). Sonic hedgehog signaling is essential for hair development. Curr. Biol. *8*, 1058-1068.

Svärd, J., Heby Henricson, K., Persson-Lek, M., Rozell, B., Lauth, M., Bergstöm, Å., Ericson, J., Toftgård, R., and Teglund, S. (2006). Genetic Elimination of Suppressor of Fused reveals an essential repressor function in the mammalian Hedgehog signaling pathway. Dev. Cell *10*, 187-197.

Vaziri Sani, F., Hallberg, K., Harfe, B.D., McMahon, A.P., Linde, A., and Gritli-Linde, A. (2005). Fate-mapping of the epithelial seam during palatal fusion rules out epithelial-mesenchymal transformation. Dev. Biol. *285*, 490-495.

Vidal, V.PI., Chaboissier, M-C., Lützkendorf, S., Mill, P., Hui, C-C., Ortonne, N., Ortonne, J-P., and Schedl. A. (2005). Sox9 is essential for outer root sheath differentiation and the formation of the hair stem cell compartment. Curr. Biol. *15*, 1340-1351.

Yuhki, M., Yamada, M., Kawano, M., Iwasato, T., Itohara, S., Yoshida, H., Ogawa, M., and Mishina, Y. (2004) BMPR1A signaling is necesary for hair follicle cycling and hair shaft differentiation in mice. Development, *131*, 1825-1833.

Zhang, X.M., Ramalho-Santos, M., and McMahon, A.P. (2001). Smoothened mutants reveal redundant roles for Shh and Ihh signaling including regulation of L/R asymmetry by the mouse node. Cell *105*, 781-792.

Table S1. Skin Phenotypes Associated with Loss of Shh Responsiveness in Different Compartments of the Developing Hair Follicle

Phenotypes	Genotypes						
	K14-Cre; Smo$^{f/f}$	*K14-Cre; Smo*$^{-/f}$	*Shhgfp-Cre; Smo*$^{f/f}$	*Shhgfp-Cre; Smo*$^{-/f}$	*K14-Cre; Shh*$^{f/f}$	*K14-Cre; Shh*$^{-/f}$	*Shh*$^{-/-}$
**Intact skin**							
Abnormal HF morphogenesis	(+)	(+) 1dpp	(–)1dpp	(–) 1dpp	(+) 1dpp	(+) 1dpp	(+) 18.5 dpc
Extrusion of follicular cells to the skin surface	(+)	(+) 1dpp	(–) 1dpp	(–) 1dpp	NdP	NdP	NdP
Conversion of the ORS into epidermis	(+)	(+) 1dpp	(–) 1dpp	(–) 1dpp	U 1dpp	U 1dpp	U 18.5 dpc
Hyperplasia of the IFE	(+)	(+) 1dpp	(–) 1dpp	(–) 1dpp	(+) 1dpp	(+) 1dpp	U 18.5 dpc
Dermal hypercellularity	(+)	(+) 1dpp	(–) 1dpp	(–) 1dpp	(–) 1dpp	(–) 1dpp	(–)18.5 dpc
Hypoplastic dc/DP	(–)	(–) 1dpp	(–) 1dpp	(–) 1dpp	(+) 1dpp	(+) 1dpp	(+) 18.5 dpc
Expansion of dc/DP at orthotopic and ectopic sites	(+)	(+) 1dpp	(–) 1dpp	(–) 1dpp	(–) 1dpp	(–) 1dpp	(–) 18.5 dpc
De novo HF formation from the IFE and preexisting follicles	(+)	(–)*1dpp	(–)* 1dpp	(–)* 1dpp	(–)* 1dpp	(–)* 1dpp	(–)* 18.5 dpc
Glandular metaplasia	(+)	(+) 1dpp	(–) 1dpp	(–) 1dpp	(–)	(–) 1dpp	(–) 18.5 dpc
**Skin grafts**							
Abnormal HF morphogenesis	(+)	(+)	(–)**	(–)**	(+)	(+)	(+)
Extrusion of follicular cells to the skin surface	(+)	(+)	(–)	(–)	NdP	NdP	NdP
Conversion of the ORS into epidermis	(+)	NdP	(–)	(–)	NdP	NdP	(+) *K17*
Hyperplasia of the IFE	(+)	(+)	(–)	(–)	(+)	(+)	(+)
Dermal hypercellularity	(+)	(+)	(–)	(–)	(–)	(–)	(–)
Hypoplastic dc/DP	(–)	(–)	(–)	(–)	(+)	(+)	(+)
Expansion of dc/DP at orthotopic and ectopic sites	(+)	(+)	(–)	(–)	(–)	(–)	(–)
De novo HF formation from the IFE and preexisting follicles	(+)	(+)	(–)	(–)	(–)	(–)	(–)
Glandular metaplasia	(+)	(+)	(–)**	(–)**	(–)	(–)	(–)

Abbreviations: dc/DP, dermal condensate(s)/dermal papilla(e); HF, hair follicle; IFE, interfollicular epidermis; ORS, outer root sheath; NdP, not determined but possible phenotypic manifestation; U; unclear due to delayed growth; (*) not applicable to de novo HF formation from the IFE at these early stages; (**), only rare follicles in grafts from ventral but not dorsal skin show the indicated phenotypic changes; (*K17*), outer root sheath phenotype determined only by histology and loss of *Keratin17* expression; 18.5 dpc, analysis up to 18.5 dpc due to lethality at birth; 1dpp, analysis up to 1 dpp due to lethality 24 hours after birth.

Table S2. Molecular Markers Used in Skin from Control and *K14-Cre; Smo$^{f/f}$* Mutant Mice

Marker	Control IFE and HF	Mutant IFE, HF and de novo-formed HF (nHF)
^{3}H-TdR (2 hours)	ORS, matrix and IFE (basal layer)<br>Dermis	ORS<br>↑ in CTS, dermis and IFE (basal and suprabasal layers)<br>↓ in the matrix
^{3}H-TdR (60 days)	LRC nested in the bulge<br>LRC in the IFE	Absence or presence of a few scattered LRC along the ORS and in the matrix<br>↑ number of LRC in IFE (basal and suprabasal levels)<br>LRSC in dc/DP/CTS<br>↑ number of LRSC in the dermis excluding the dc/DP/CTS<br>Cells of dc that diluted the label
**Histochemistry**		
Alkaline phosphatase	dc/DP	Expanded dc/DP associated with HF and nHF (*)<br>dc/DP embedded in follicular epithelium (*)<br>dc/DP embedded in the IFE<br>Similar changes in dorsal and ventral skin
**Immunohistochemistry**		
Activated caspase 3	Anagen HF: some IRS and ORS cells	No alterations
β-catenin	Cell-cell borders in the IFE<br>Nuclear in differentiating cortical cells of the HS<br>Nuclear in secondary hair germ cells at the telogen-to-anagen transition	↑ at cell-cell borders of IFE after 15dpp<br>↑ at cell-cell borders in the follicular epithelium<br>↓ numbers of differentiating HS cells with nuclear staining<br>Nuclear in a subset of dc cells associated with HF and nHF
E-cadherin	IFE<br>ORS<br>Matrix and its progeny (weak)	IFE<br>All layers of the hyperplastic ORS<br>E-cadherin-negative matrix cell aggregates among epidermal keratinocytes (*)
Keratin 1	IFE (suprabasal layers)	IFE<br>Thickened ORS
Keratin 6	CL of the ORS	Thickened CL of the ORS<br>Some areas of the IFE
Keratin 8	Merkel cells (Haarscheiben epidermis of guard HF)	No alterations in dorsal skin
Keratin 14	IFE (basal layer)<br>ORS	All layers of IFE<br>All layers of the hyperplastic ORS (*)
Keratin 15	Cells in the bulge of all HF at telogen	Absent in many follicles<br>Absent in cysts<br>Some scattered cells in the ORS and matrix of a few follicles

Ki 67	Some cells of the ORS<br>Matrix<br>Dermis	Normal in ORS<br>↓ in the matrix<br>↑ number of Ki 67-positive cells in the dermis, CTS and epidermis<br>Ki 67-positive cell aggregates extruded at the skin surface
P-Smads1, 5, 8	CL of the ORS<br>Some cells of the HS and IRS	Some P-Smad-positive cells at the skin surface (early stages)<br>Reduced domain in the epithelium of large HF<br>In a subset of cells of dc
Shh	In a subset of matrix cells at anagen<br>Diffusion to the DP/lower ORS	Expanded domain in the matrix (*)<br>Shh-positive aggregates among epidermal keratinocytes (*)<br>Shh-positive cells at the skin surface (*)<br>nHF (early and late development)
Syndecan 1	ORS (weak)<br>IFE<br>DP	Strong in all layers of the expanded ORS<br>Strong in the IFE<br>dc/DP associated with HF and nHF
ZO-1	IFE<br>IRS<br>ORS (weak)	IFE<br>All layers of the thickened ORS<br>Extruded matrix cells are ZO-1-negative
**In situ hybridization**		
Bmp2	Hair placode at 14.5 dpc	No alterations (*)
Bmp4	dc at early stages<br>DP, upper matrix, HS precurors and CL at anagen	dc/DP of all HF and nHF<br>Reduced domain in the epithelium<br>Cells located at the skin surface (extrusion) (*)
Cyclin D1	ORS and matrix<br>Dermis (weak)	Normal in ORS<br>↓in the matrix<br>↑in the dermis
Dct/*Trp2*	MSC in the bulge<br>Amplifying progeny in the upper matrix (melanoblasts)<br>1-2 melanoblasts in the lower ORS	Scattered *Dct*-positive cells in the ORS<br>↓number of *Dct*-positive cells in the matrix of all HF and nHF<br>Numerous *Dct*-positive cells in the dermis
Epiprofin	IRS	Reduced domain of expression
Foxe1	ORS	Severely reduced to background levels during both early and late developmental stages
Gli1	Early stages: HF placode/germ and dc<br>Anagen: hair matrix, IRS, HS precursor cells, lower ORS and DP	Reduced to background levels at all stages in the follicular epithelium (*)<br>Increased in dc/DP and dermis underlying the IFE (*)
Gli2	Similar to *Gli1*, but weaker labeling	No alterations(*)
Gli3	Similar to *Gli1*, but weaker labeling	No alterations(*)
Hoxc13	Matrix cells<br>Differentiating HS cells	Reduced expression levels<br>Reduced domain of expression

K17	ORS and its CL<br>Differentiating HS cells	Absent in ORS (* and **)<br>Present in the expanded CL (* and **)<br>Present in HS cells (* and **)<br>Ectopic expression in the IFE suprabasally (* and **)
Msx1/*Msx2*	Follicular matrix<br>Early differentiating IRS/HS cells	Reduced expression levels (*)<br>Reduced domain of expression (*)<br>Follicular keratinocytes at the skin surface
Ptc1	Expression patterns similar to those of *Gli1*	Similar alterations to those found with the *Gli1* probe (*)
Shh	In a subset of matrix cells at anagen	Expanded domain in the matrix (*)<br>*Shh*-positive aggregates among epidermal keratinocytes (*)<br>*Shh*-positive cells at the skin surface (extrusion) (*)<br>"Placode" and matrix of nHF
Smo	In HF epithelium and mesenchyme	No alteration (the probe hybridizes with the full-length transcripts)
Sox9	ORS and bulge	Reduced to background levels during early and late developmental stages
Wnt5a	In dc/DP at early stages of HF morphogenesis<br>At anagen: in ORS and some cells of the IRS, absent in DP	Reduced domain in IRS<br>Absent in ORS<br>Follicular keratinocytes at the skin surface (extrusion)<br>↑in dc associated with HF and nHF
Wnt7b	IFE	IFE<br>All layers of the thickened ORS
Wnt10b	Follicular matrix	Follicular matrix<br>"Placode" and matrix of nHF

	**<u>Orthotopic mammary glands (early postnatal stages, except ♦)</u>**	**<u>Heterotopic glands (Hg) and orthotopic mammary glands (early postnatal stages)</u>**
**Immunohistochemistry**		
Keratin 8	Luminal cells	Luminal cells (*)
Shh/Ihh	Negative	Negative (*)
Keratin 6	Luminal cells	Luminal cells
Keratin 14	Luminal cells	Luminal cells
Smooth muscle α-actin	Myoepithelial cells	Myoepithelial cells
**In situ hybridization**		
β-casein	Alveoli (lactation day 1) ♦	Hg treated in vitro with a lactogenic cocktail
Barx2	luminal cells	luminal cells
Gli1	No expression	No expression
Ihh	No expression	No expression
Ptc1	No expression	No expression

Shh	No expression	No expression
Sox9	Luminal cells	Luminal cells
Tbx3	Luminal cells	Luminal cells
Wnt7b	Luminal cells	Luminal cells
Wnt10b	Luminal cells	Luminal cells
	**Mammary gland buds in control embryos**	**Mammary gland buds in *hK14-Shh* embryos**
Wnt10b	Strong	Present in abnormal buds **Δ**
PTHrP	strong	Expressed in abnormal buds **Δ**<br>↑ expression levels in the epidermis
Tbx3	Strong	Reduced expression in abnormal buds **Δ**<br>↑ expression levels in the dermis

Abbreviations. CL, companion layer of the outer root sheath; CTS, connective tissue sheath of the hair follicle; dc, dermal condensate (s); DP, dermal papilla (e); HF, hair follicle; IFE, interfollicular epidermis; IRS, inner root sheath; LRC, label-retaining cells; LRSC, label-retaining stromal cells; MSC, melanocyte stem cells; nHF, de novo-generated HF from the IFE and from pre-existing follicles; ORS, outer root sheath; ↑, increase (d); ↓, decrease (d); *, similar expression patterns and morphological features in *K14-Cre; Smo*$^{-/f}$ embryos and/or new-borns and/or skin grafts; **, similar expression patterns in skin grafts from Shh$^{-/-}$ fetuses; **Δ,** *hK14-Shh* embryos display either a total lack of mammary gland buds or presence of abnormal buds.

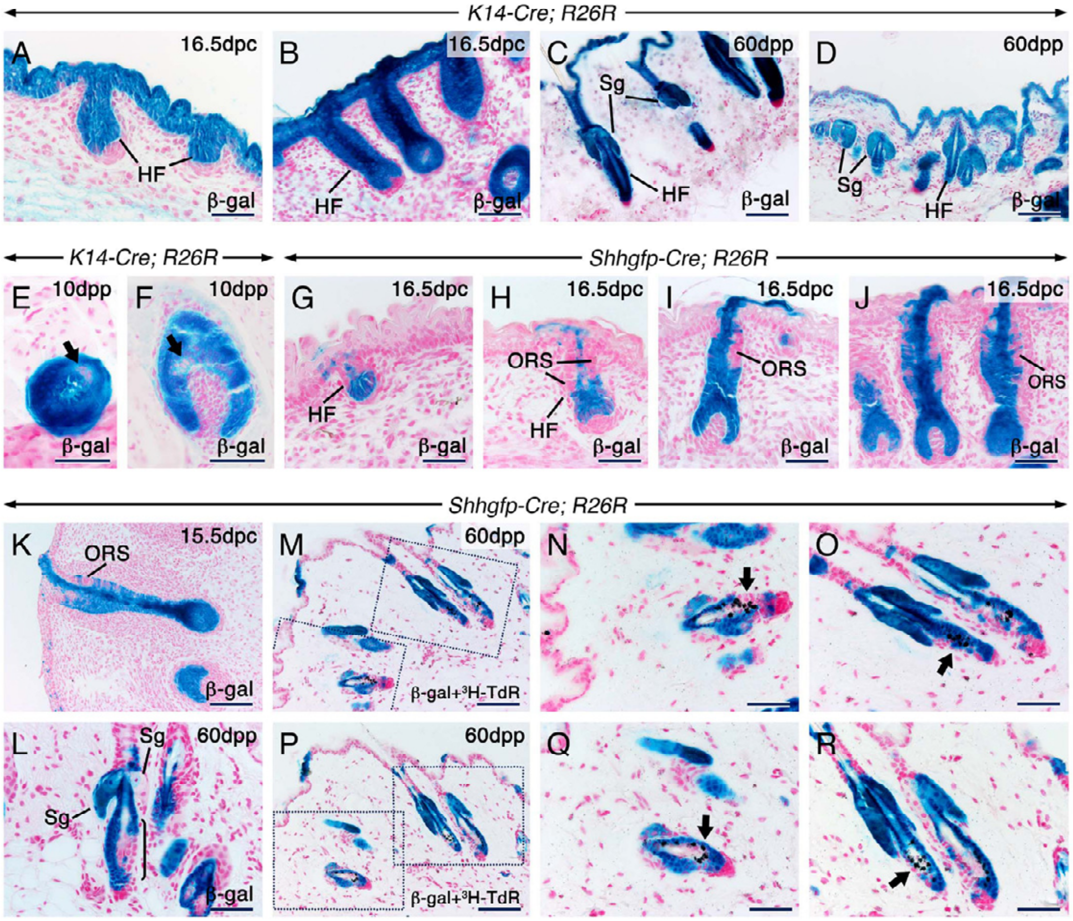

Figure S1. Differences between K14-Cre- and Shhgfp-Cre-Mediated Recombination Events at the *R26R* Reporter Locus in the Skin

β-galactosidase staining (β-gal) showing the sites of K14-Cre- and Shhgfp-Cre-mediated recombination events at the *R26R* reporter locus and ^{3}H-TdR autoradio-graphy showing hair follicle label retaining stem cells (LRC) in the bulge niche. (A-F) Skin sections from *K14-Cre; R26R* mice showing β-gal staining (blue) in sebaceous glands (Sg), hair follicles (HF) and epidermal keratinocytes. Panels (D)-(F) show thinner (4 µm) skin sections than the ones shown in panels (A)-(C) which are 6 µm-thin. (E and F) *lacZ*-negative cells are present in a domain encompassing that of melanocytes (arrows). (G-R) Sections from *Shhgfp-Cre; R26R* mice. (G-K and L) β-gal staining. Both pelage hair (G-I) and whisker (J and K) follicles display a subset of *lacZ*-negative cells. (I, J and K) Follicles with *lacZ*-negative cells in the outer root sheath (ORS). (L) The bulge niche (bracket) of a HF at telogen comprises both *lacZ*-positive and *lacZ*-negative cells. Also shown in (L) a HF with a *lacZ*-positive and a *lacZ*-negative sebaceous glands (Sg). (M-R) Sections of skin biopsies taken 60 days after ^{3}H-TdR injections, stained for β-gal and processed for autoradiography. LRC contain numerous silver grains and thus appear black on sections. The sections shown in (M-O) are serials to those shown in (P-R). (N) and (O) are high magnification views of (M). (Q) and (R) are high magnification views of (P). Serial sections showing that follicles in which the bulge region appears to contain only *lacZ*-positive-LRC, actually contains also *lacZ*-negative-LRC (arrows in [N, O, Q and R]). Scale bars: 50 µm (A-C, E-J, L, N, O, Q, R) and 100 µm (D, K, M, P).

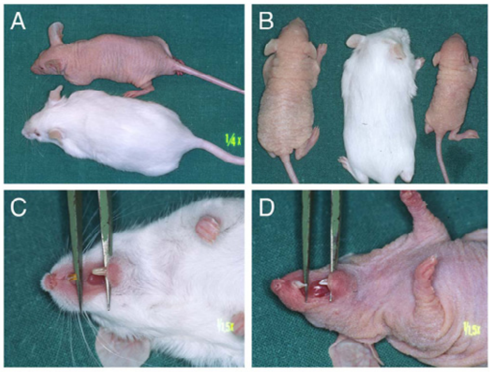

Figure S2. ***K14-Cre; Smo*$^{f/F}$ Mice Are Totally Devoid of Hair**

(A, C and D) *K14-Cre; Smo*$^{f/f}$ mutant and control mice at 3 months of age. Note the total lack of hairs and wear of incisors in the mutant. (B) Two month-old mutant and control mice. The mutant on the right is growth-delayed and was not included in this study.

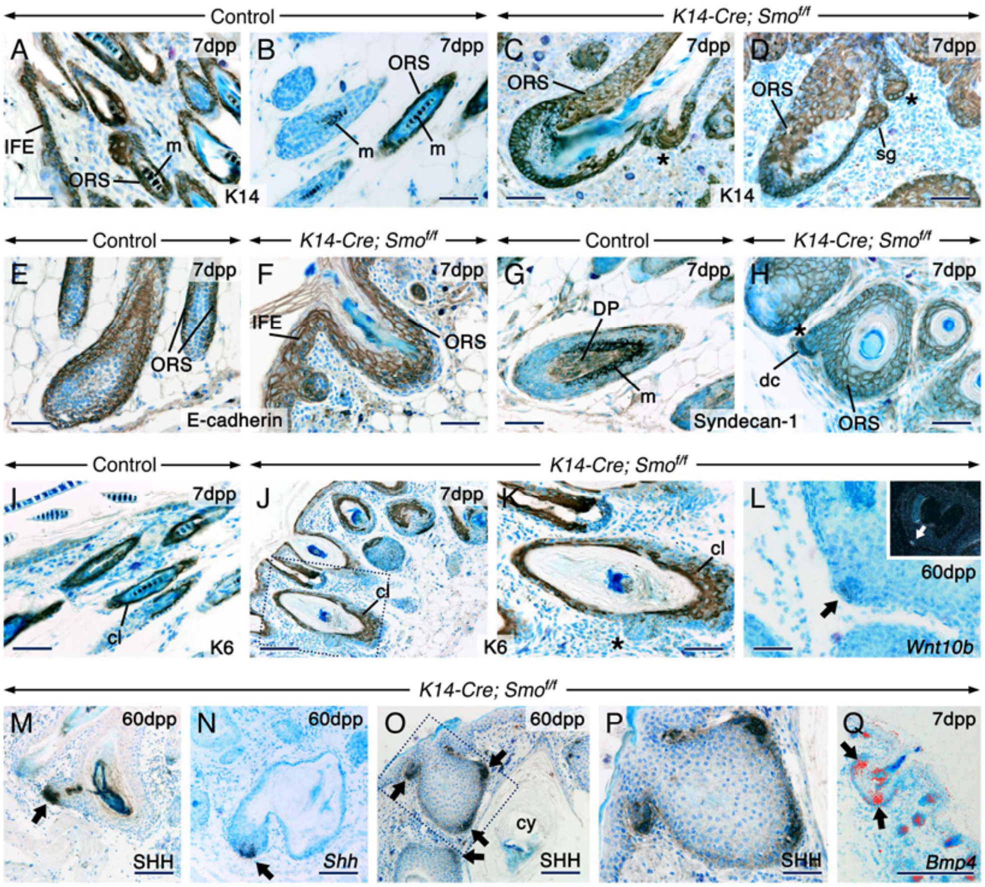

Figure S3. Hyperplasia of the Epidermis and Outer Root Sheath and De Novo Hair Follicle Formation in the Absence of Epithelial Smo Function

Immunohistochemistry (A-K, M, O, P) and in situ hybridization (L, N, Q) of sections from control (A, B, E, G, I) and *K14-Cre; Smo$^{f/f}$* (C, D, F, H, J-Q) dorsal (A-P) and ventral (Q) skins. Immunolabeled sites appear brown and in situ hybridization signals appear as bright (inset in [L]), black (L and N) or red (Q) grains. (A-H) K14, E-cadherin and syndecan-1 stainings highlight the hyperplastic outer root sheath (ORS) and interfollicular epidermis (IFE**)** in the mutant skin. (I-K) Keratin 6 (K6) staining shows a hyperplastic companion layer (cl) in mutant as compared to control follicles. Early stages of de novo hair follicle formation from pre-existing follicles (asterisks in [C, D, H and K]). (H) A syndecan-1-positive dermal condensate (dc) underlying a de novo follicle. (L-Q) The arrows mark de novo follicles emerging from pre-existing follicles and expressing *Wnt10b* (L), Shh protein (M, O, P), *Shh* (N) and *Bmp4* (Q). Inset in (L) is a low magnification, dark field view of (L). cy, cyst; DP, dermal papilla; m, melanin pigment; sg, sebaceous gland. Scale bars: 50 µm (A-I, K, L, P), 100 µm (J, M, N, O) and 200µm (Q).

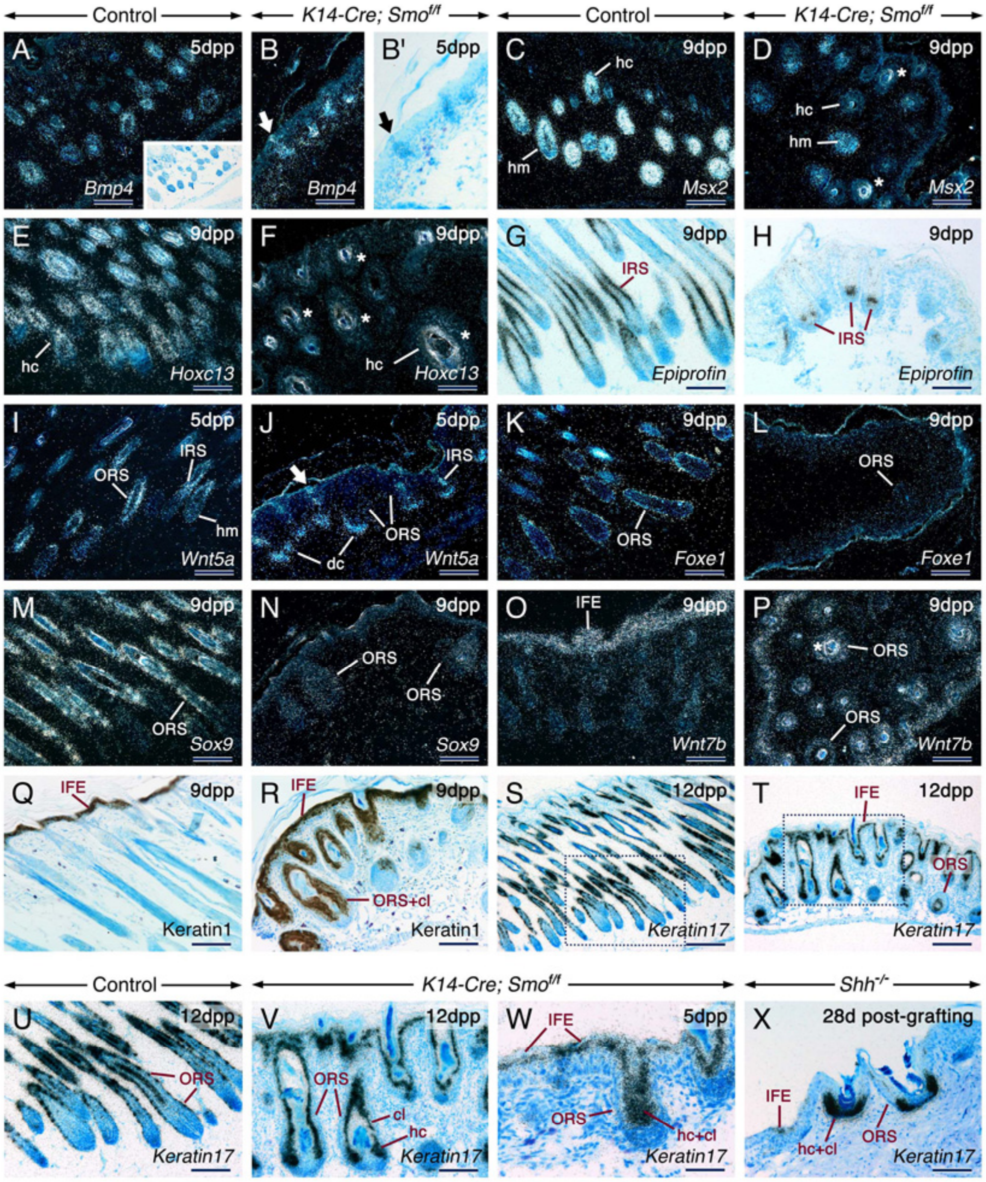

Figure S4. Extrusion of Follicular Cells and Altered Inner Root Sheath, Hair Shaft and Outer Root Sheath Development in the Absence of Epithelial Smo Activity

In situ hybridization (A-P and S-X) and immunohistochemistry (Q and R) on sections from control (A, C, E, G, I, K, M, O, Q, S, U) and *K14-Cre; Smo*$^{f/f}$ (B, B', D, F, H, J, L, N, P, R, T, V, W) skins. Section of a skin graft from a *Shh*$^{-/-}$ fetus at 28 days post-grafting (X). Dark field images (A, B, C-F, I-P). Bright field images (inset in A, B', G, H, S-X). Extrusion of follicular keratinocytes (arrows) expressing *Bmp4* (B and B') and *Wnt5a* (J) at the skin surface of mutants. In addition, the mutant skin displays the following anomalies: decreased levels and domains of expression of *Msx2* (D) and *Hoxc13* (F) in the hair matrix (hm) and differentiating hair cells (hc), a reduced domain of *Epiprofin* (H) expression in the inner root sheath (IRS), increased *Wnt5a* expression in the dermal condensates (dc) and severely reduced *Wnt5a expression* in the outer root sheath (ORS) in (J), severe reduction to background levels of *Foxe1* (L), *Sox9* (N) *and Keratin 17* (V

and W) expression in the ORS, ectopic expression of *Wnt7b* (P) and Keratin 1 (R) in the ORS and ectopic *Keratin17* expression in the interfollicular epidermis (IFE) at suprabasal levels. However, in the mutants, the companion layer (cl) of the ORS and hc show normal expression levels of *K17* (T, V, W). *Keratin 17* expression is severely reduced in the ORS and is expressed ectopically in the IFE of a *Shh*$^{-/-}$ skin graft (X). The asterisks in (D), (F) and (P) indicate artefactual shiny areas generated by tissue distorsion and cellular debris. Scale bars: 50 µm in (W), 100 µm (A-R, U, V, X) and 200 µm (S, T).

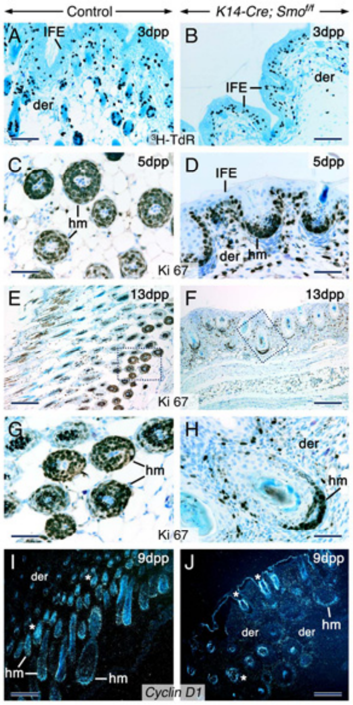

Figure S5. Reduced Cell Proliferation in the Hair Matrix and Increased Dermal and Epidermal Proliferation in the *K14-Cre; Smo*$^{f/f}$ Skin

Skin sections from postnatal controls (A, C, E, G, I) and mutants (B, D, F, H, J). (A and B) Sections from skin biopsies taken 2 hours after ^{3}H-TdR injection were processed for autoradiography. Cycling cells appear black. (C-H) Immunohistochemistry with anti-Ki 67 antibody (dark brown). (I and J) In situ hybridization for *Cyclin D1*. The asterisks in (I and J) indicate shiny artefacts due to tissue distorsion and cellular debris. der, dermis; IFE, interfollicular epidermis; hm, hair matrix. Scale bars: 50 µm in (C, D, G, H), 100 µm (A, B) and 200 µm (E, F, I, J).

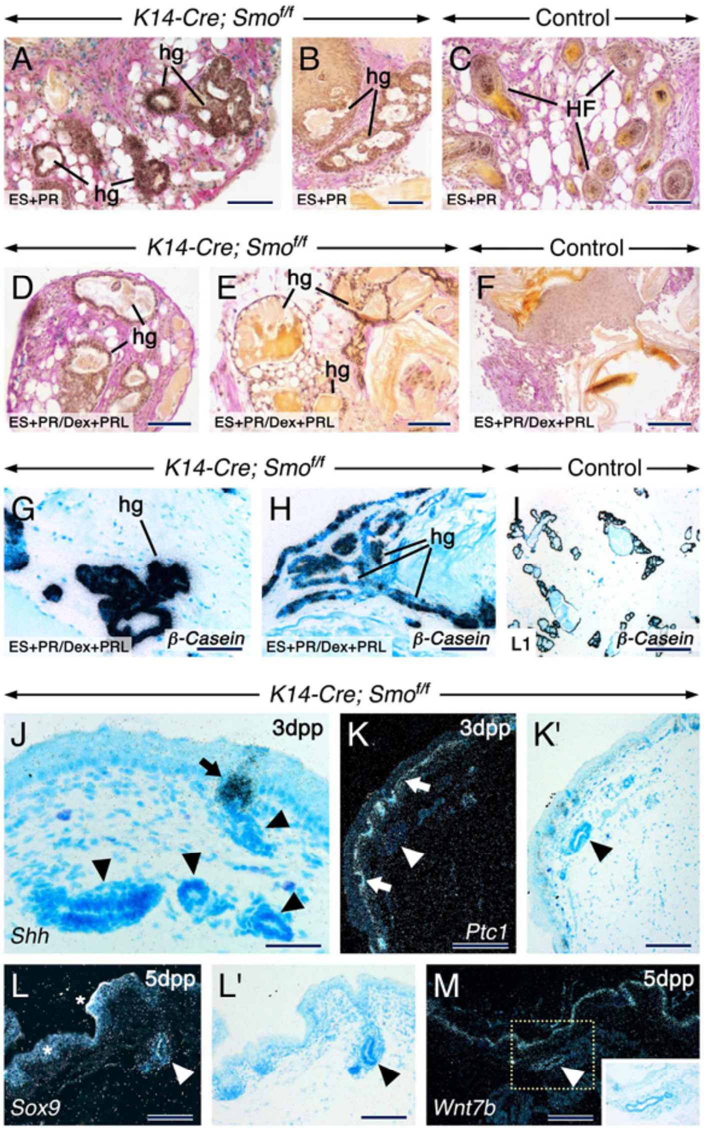

Figure S6. The Heterotopic Glands Differentiate into Mammary Glands In Vitro and Express Mammary Gland Markers In Vivo

Sections from a dissected heterotopic gland (A) and mid-ventral skin explants (B-H) from 3 dpp (A-D and G) and 4 dpp (E, F, H) *K14-Cre; Smo$^{f/f}$* mutant (A, B, D, E, G, H) and control (C and F) mice cultured in vitro and stained with alcian blue van Gieson (A-F) or hybridized with a *β-Casein* probe (G and H). Hybridization signal appears as black grains. (A-C) Explants cultured for 4 weeks as indicated in Experimental Procedures, the last 2 weeks included treatment with 17-β-estradiol (ES) and progesterone (PR). (A and B) The mutant explants contain well-developed heterotopic glands (hg). (C) Only hair follicles (HF) are present in the control explant. (D and G) Hg cultured for 4 weeks, the 2nd and 3rd weeks included treatment with ES and PR and during the last week the above hormones were replaced by dexamethasone **(**Dex**)** and prolactin **(**PRL**)**. The hg develop wide lumina filled with secretory material (D) and express *β-Casein* (G). (E, F and H) The explants were mixed with cleared mammary fat pads and cultured for 4 weeks as in (D and G). In the mutant explants the hg show wide lumina with secretory products (E) and express *β-Casein* (H). Absence of hg in an explant from a control mouse (F)._(I)

Section from a control orthotopic mammary gland at lactation day1 (L1) showing *β-Casein* expression. (J-M) In situ hybridization of sections from from *K14-Cre; Smo*$^{f/f}$ mid-ventral skin. Bright field (J, K', L') and dark field (K, L, M) images. (J) *Shh* is expressed in hair follicles (arrow), whereas the hg (arrowheads) are devoid of *Shh* transcripts. (K-M) The hg (arrowheads) are devoid of *Ptc1* transcripts (K, K') but express *Sox9* (L, L') and *Wnt7b* (M). The arrows in (K) and (K') show *Ptc1* expression in the mesenchyme. Inset in (M) is a bright field image of the indicated area. Scale bars: 50 µm (J), 100 µm (A-H), 200 µm (K-M) and 500 µm (I).

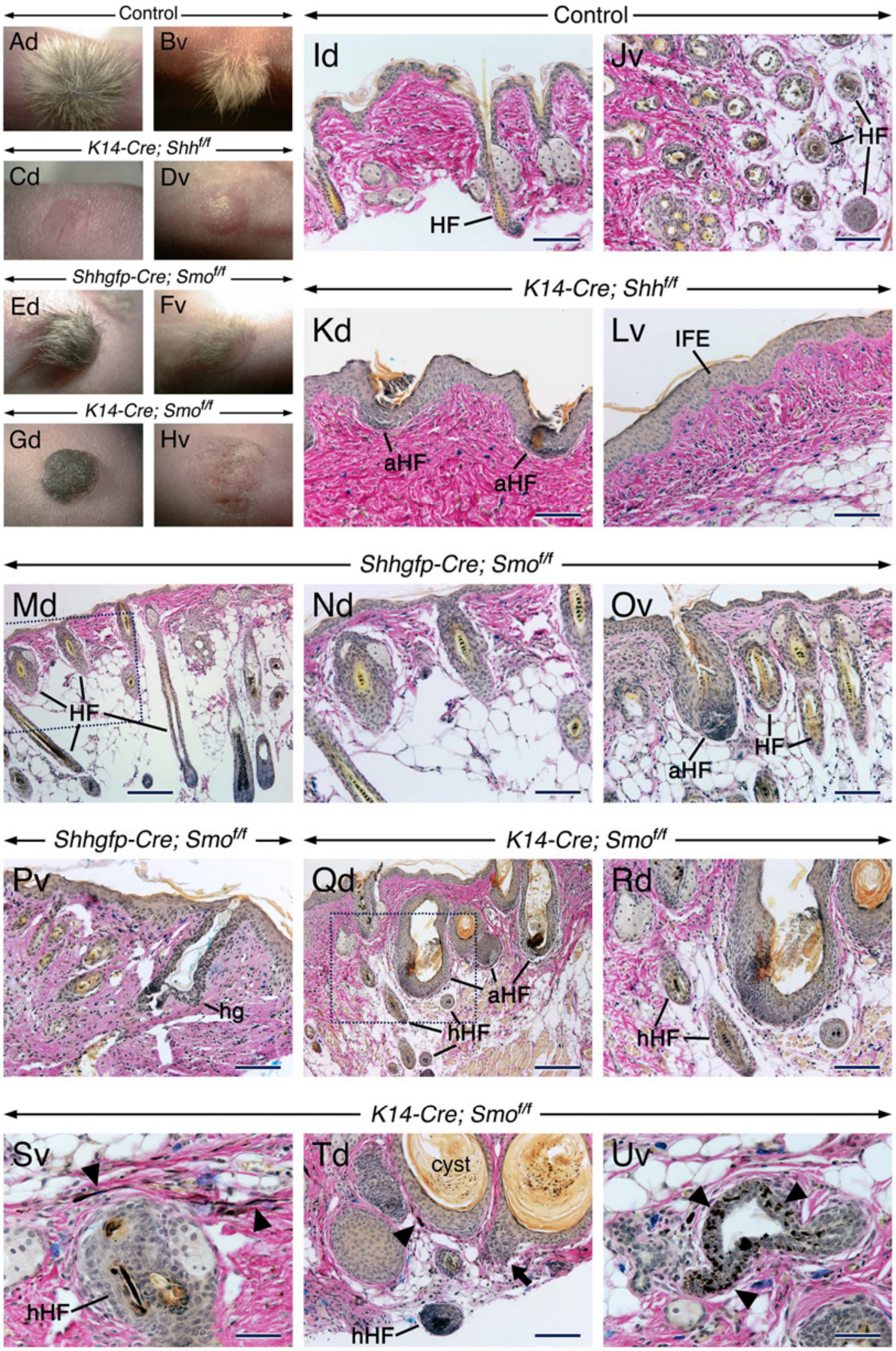

Figure S7. Glandular Metaplasia and De Novo Follicular Formation Do Not Occur in the *Shh* Null Skin. Some Glands Develop in the *Shhgfp-Cre; Smo*$^{f/f}$ Skin

Skin biopsies were taken from 18.5 dpc fetuses and grafted onto recipient back skin sites of *nu/nu* mice and analysed at 4 weeks post-surgery. External aspect of grafts from dorsal

(Ad, Cd, Ed, Gd) and ventral (Bv, Dv, Fv, Hv) skins. A dense fur developed in the grafts from control fetuses (Ad and Bv). The grafts from *K14-Cre; Shh*$^{f/f}$ (Cd and Dv) and *K14-Cre; Smo*$^{f/f}$ (Gd and Hv) fetuses are devoid of hair. The doesal skin graft (Ed) of a *Shhgfp-Cre; Smo*$^{f/f}$ fetus formed a dense fur, whereas the ventral graft (Fv) generated a less dense fur than the ventral graft from controls (Bv). Sections from dorsal (Id, Kd, Md, Nd, Qd, Rd, Td) and ventral (Jv, Lv, Ov, Pv, Sv, Uv) skin grafts stained with alcian blue van-Gieson. (Id, Jv) Sections of control grafts showing normal hair follicles (HF). (Kd and Lv) Sections of *K14-Cre; Shh*$^{f/f}$ grafts showing abnomal hair follicles (aHF), hyperplasia of the interfolliculae epidermis (IFE) and absence of de novo hair follicle formation and glandular metaplasia. (Md, Nd, Ov, Pv) Sections of *Shhgfp-Cre; Smo*$^{f/f}$ grafts showing normal follicular development in the dorsal skin (Md and Nd). The ventral skin (Ov and Pv) displays some aHF and heterotopic glands (hg). (Qd, Rd, Sv, Td, Uv) Sections of *K14-Cre; Smo*$^{f/f}$ grafts showing aHF. The dermis shows ectopic melanocytes (arrowheads in [Sv] and [Td]), some of which colonized the host albino *nu/nu* follicles (hHF) and resulted in their pigmentation (melanin granules appear black). Note the de novo-generated from a pre-existing follicle (arrow in [Td]). Arrowheads in (Uv) indicate melanin-containing cells (black) intercaled between cells of a heterotopic gland. (Nd) and (Rd) are high magnification views of (Md) and (Qd), respectively. Scale bars: 50 µm (S, U), 100 µm (I-L, N-P, R, T) and 200 µm (M, Q).

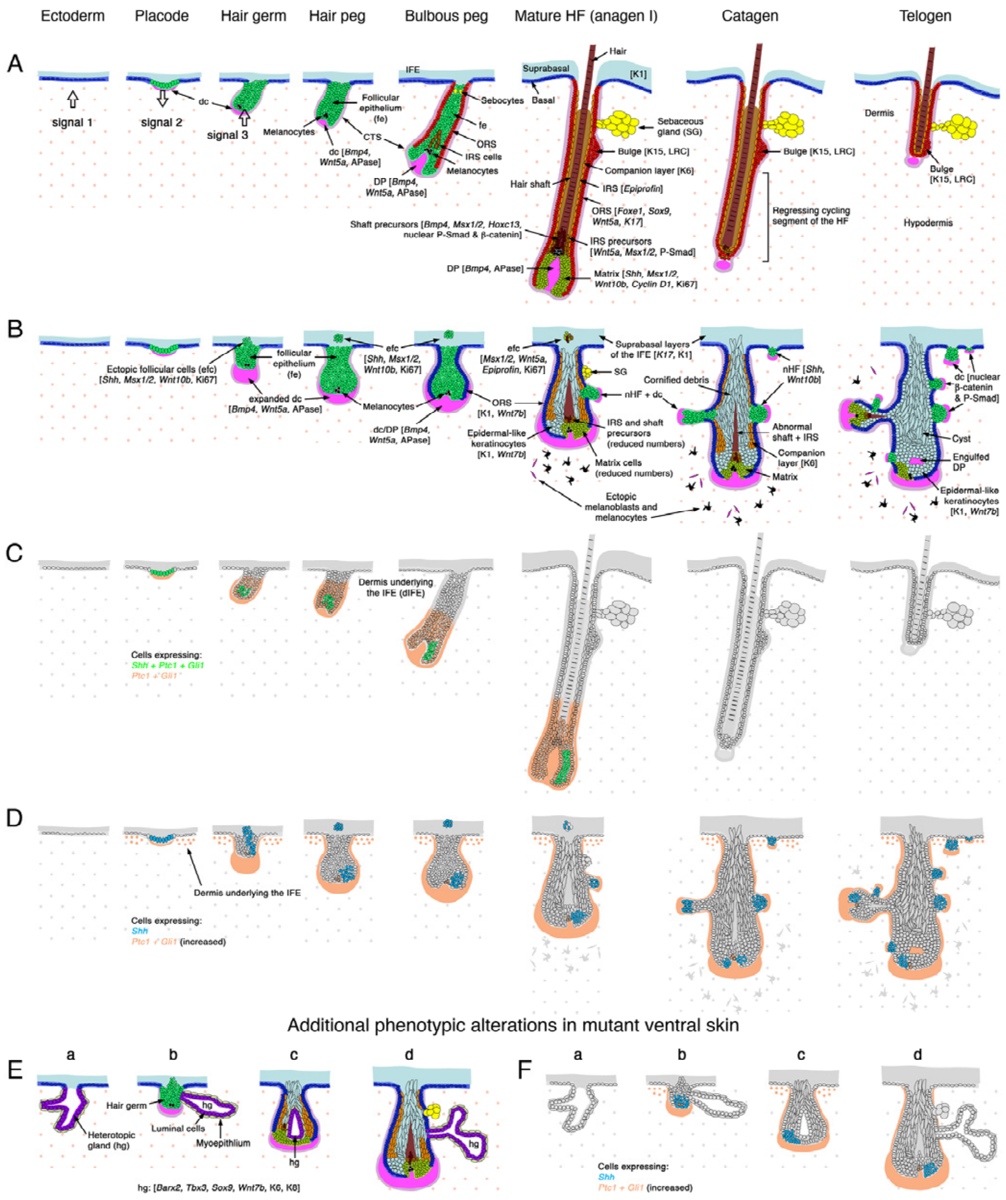

Figure S8. Schematic Diagrams Showing the Phenotypic Consequences and Signaling Alterations Following K14-Cre-Mediated Removal of Smo in the Skin

(A and B) Schematics showing the different stages of hair follicle (HF) development and follicles at the catagen and telogen phases in control (A) and *K14-Cre; Smo*$^{f/f}$ (B**)** skins. **(**A**)** Hair follicular fate is induced in the basal layer of the ectoderm by a putative dermal signal (signal 1), the hair placode induces (signal 2) dermal cells to aggregate into dermal condensates (dc, magenta). The dc are thereafter encased by the follicular epithelium as dermal papillae (DP). Hair follicle (HF) growth, morpho-genesis and differentiation depend on messages (signal 3) from the dc/DP and on intraepithelial signals. The hair matrix (dark green) at the base of the mature follicle (anagen 1) consists of highly proliferating cells. Upon differentiation, matrix cells generate the different layers of differentiating cells of the inner root sheath (IRS, light brown) and hair shaft (dark

brown). The outer root sheath (ORS, red) is continuous with the basal layer of the interfollicular epidermis (IFE, dark blue).The companion layer (orange) of the ORS separates it from the IRS. The bulge region, home of follicular stem cells, is situated under the sebaceous gland (SG, yellow). The neural-crest-derived melanocytes (black) and their progenitors colonize the follicular epithelium. In the mature HF, melanocyte stem cells populate the bulge/sub-bulge (not represented) while their amplifying progeny and differentiated melanocytes are located in the upper matrix. The entire HF is surrounded by cells of the connective tissue sheath (CTS, violet). During catagen, the HF undergoes a progressive destructive phase culminating in the removal of its lower two-thirds (cycling segment). After completion of catagen, the permanent segment of the HF enters the resting telogen phase. Subsequently, a new HF formative phase is initiated following proliferation of follicular stem cells in response to signals from the DP (not represented). Cells of the suprabasal layers of the epidermis, dc, DP and CTS are not represented. The outlines of these structures are indicated by colors. Cells of the dermis and hypodermis are indicated by dots. (B) The *K14-Cre; Smo*$^{f/f}$ skin shows defects in HF, IFE and their associated mesenchymal cells. Mutant HF undergo abnormal morphogenesis and differentiation as a consequence of loss and lower production of follicular cells (*Shh-*, *Msx1/-2*, *Bmp4-*, *Wnt5a-*, *Epiprofin-* and Ki67-positive), conversion of the ORS (blue) into epidermis (loss of *Foxe1*, *Sox9* and *Keratin 17* [*K17*] expression, acquisition of *Wnt7b* and Keratin 1 [K1] expression and production of epidermal-like keratinocytes[light blue]), absence of development of a follicular stem cell niche and an impressive expansion of alkaline-phosphatase (APase)-positive dc/DP. The latter two defects are responsible for induction of de novo HF formation (nHF) from pre-existing follicles. The number of IRS and shaft precursors with nuclear β-catenin and P-Smad staining is decreased, whereas β-catenin protein accumulates at cell-cell borders in the HF epithelium and IFE (not represented). A subset of cells in the dc/DP shows peculiar nuclear β-catenin and P-Smad accumulation. As development proceeds, the mutant follicles convert into epidermal cysts filled with cornified debris (light blue). The IFE is hyperplastic, expresses *Keratin 17* suprabasally and generates de novo HF (nHF) in response to mesenchymal cells. The rest of the dermis is hypercellular and contains ectopic melanocytes (black) and their progenitors (magenta). Only rare mutant follicles enter the telogen phase (not represented). (C and D) Shh signaling in control (C**)** and *K14-Cre; Smo*$^{f/f}$ (D) skins. Cells producing and responding to Shh (green). Cells responding to Shh (orange) and cells producing Shh (cyan; only in the mutants). (C) In control skin, Shh is expressed in a subset of follicular epithelial cells and signals both the HF epithelium and its associated mesenchyme (dc/DP/CTS). (D) Removal of Smo in the follicular epithelium results in abrogation of Shh responsiveness in the epithelium and increased orthotopic and ectopic Shh signaling (due to Shh protein spillover) in the dc/DP/CTS and in the dermis underlying the IFE (dIFE), respectively. **(**E**)** In the mutant ventral skin, in addition to the alterations shown in (B), some HF convert partially or totally into heterotopic glands (hg, violet) as a result of loss of epithelial Shh responsiveness and mesenchymal influences. (a**)** A hg that developed from the IFE at the expense of a HF. (b) A hg that developed from a hair germ. (c) A hg that developed from the hair matrix. (d) A hg that developed from the ORS. The hg express mammary-gland markers including *Barx2*, *Tbx3*, *Wnt7b* and *Sox9*, keratin 6 (K6) and keratin 8 (K8). (F**)** Shh signaling in the ventral skin. The hg and are devoid *of Shh/Ihh, Ptc1* and *Gli1* expression. Additional abbreviations: efc, ectopic follicular cells; K15, Keratin 15; LRC, label-retaining cells.
